# Beyond the convergence of metabolic reprogramming in primary liver cancer: a comprehensive review on energy and lipids metabolism

**DOI:** 10.3389/fcell.2026.1890663

**Published:** 2026-08-03

**Authors:** Galina S. Stupnikova, Igor A. Popov, Stanislav I. Pekov

**Affiliations:** Laboratory for Molecular Medical Diagnostics, Moscow Institute of Physics and Technology, Dolgoprudny, Russia

**Keywords:** cholangiocarcinoma, glycolysis, hepatocellular carcinoma, lipid metabolism, metabolic reprogramming, primary liver cancer

## Abstract

Hepatocellular carcinoma and cholangiocarcinoma, the most common primary liver cancers, are usually considered quite different pathologies. However, convergent metabolic reprogramming across different progenitor cells can result in similar molecular alterations and, even in a combined form of cancer that is characterized by transitional features and a shared phenotype. In this review, we summarize essential steps in glucose and lipid metabolism to distinguish similarities in glucose and energy metabolism reprogramming from divergent lipid remodeling in different types of primary liver cancers. We show the convergent nature of metabolic alterations in glucose decomposition and related mitochondrial enzymes. Also, we outline the essential role of lactate in promoting cell viability, adaptation to increased biomass synthesis, and fueling surrounding cancer cells to support their growth and proliferation. Lipid metabolism, in contrast, was found to be dramatically different between primary liver cancers. Hepatocellular carcinoma relies on *de novo* fatty acids synthesis, for which mitochondrial activity shifts from energy production to citrate efflux. Cholangiocarcinoma, in contrast, relies on fatty acids uptake from the extracellular space and, at later stages, even engages in beta-oxidation, which is uncharacteristic of hepatocellular carcinoma. This yields an altered lipid portrait for these pathologies despite the overall convergent alterations in energy metabolism. With this review, we provide not only fundamental insights for further primary liver tumor metabolism investigation, but also an emphasis on the independence of lipid alterations from energy metabolism reprogramming, vital for further basic and translational applications of metabolomics and lipidomics to a broad range of cancers.

## Background

Primary liver cancers (PLCs) are the sixth most common newly diagnosed cancers worldwide. Globally, incidence rate and mortality are over two times higher among men than among women—it is the second leading cause of cancer deaths among males (Sung et al., 2021). PLCs comprise hepatocellular carcinoma (HCC, 80% cases), cholangiocarcinoma (CCA, 15% cases), and other rare subtypes (Rumgay et al., 2022; Cardinale, 2019). HCC and CCA, the most common types of PLC, have similar lists of risk factors, but their influence on the disease onset is different. Virus infections of hepatitis B or C, heavy alcohol intake, and metabolic conditions are primary risk factors for HCC (Feng and Zhao, 2024; Llovet et al., 2021). Liver flukes, cholangitis, and stones are the primary risk factors for CCA, while metabolic conditions, excessive alcohol consumption, and hepatitis viruses play lesser, but still not neglectable roles (Khosla et al., 2024; Dolbnya et al., 2024). The dissimilar etiology of HCC and CCA stems from their progenitor cells. While HCC originates from hepatocytes, CCA is derived from bile duct epithelial cells. At the molecular level, both cancers are characterized by frequent TP53 mutations, but alterations in the TERT promoter and CTNNB1 are typical for HCC (Wang et al., 2024; Calderaro et al., 2019), while BRAF, EGFR, KRAS, and IDH mutations are common in CCA (Dorovatovskaia et al., 2025; Andersen et al., 2012). Surgery, including liver transplantation, remains the cornerstone of resectable PLCs treatment (Orcutt and Anaya, 2018) and many efforts are made to improve the accuracy of resection and help in decision-making during the surgery (Giordano et al., 2020; Zhang et al., 2021; Hakoda et al., 2022). The molecular basis of such methods is not directly linked with genetic alterations, but with metabolic transformation accompanying malignancy—the high proliferation rate of tumor cells requires increased energy production and biomass synthesis, while hypovascularization limits the nutrient supply. Thus, glucose and lipid metabolism in cancer cells turns out to be significantly altered compared to paracarcinoma tissues, which can be used pre- and intra-operatively to differentiate healthy and malignant tissues accurately (Berkemeyer, 2023; Wang et al., 2023; Li Z. et al., 2017; Pekov et al., 2025).

Treatment strategies for unresectable PLCs, as well as adjuvant therapy for resectable ones, differ between HCC and CCA (Feng and Zhao, 2024; Llovet et al., 2021; Khosla et al., 2024; Shannon et al., 2022), which should place these nosologies onto the opposite sides of the PLC spectrum. Still, there exists a relatively rare variant of liver cancer—combined hepatocellular-cholangiocarcinoma (cHCC-CCA, up to 5% cases), which presents as a solitary mixture of HCC and CCA components and, sometimes, transitional features (Li et al., 2024a; Calderaro et al., 2023). The tumor can consist of neighboring regions with HCC and CCA phenotype, intermingled cells of both types, or uniform small cells that look intermediate between hepatocytes and cholangiocytes (Wege et al., 2024). In addition to morphological characteristics, cHCC-CCA shares molecular features with HCC and CCA (Paradis and Zucman-Rossi, 2023; Zhang et al., 2026), which makes these cancers closer to each other than might be expected. Thus, a comprehensive investigation of the carcinogenesis process in the liver will benefit from considering both HCC and CCA. Healthy hepatocytes and cholangiocytes possess distinct functional specializations that govern systemic metabolism and bile transport, respectively. Consequently, when exposed to stress, such as nutrient deprivation or oncogenic transformation (Zhang et al., 2026), their baseline lineage programs drive them to adapt differently, ultimately leading to the distinct metabolic divergence observed between HCC and CCA. Thus, in this review, we will summarize current knowledge on glucose and lipid metabolism in hepatocellular carcinoma and cholangiocarcinoma to indicate both similar and unique features for these nosologies.

Reprogramming of energy metabolism is a distinctive feature of cancer (Hanahan and Weinberg, 2011). Depending on the tumor microenvironment, the availability of essential nutrients, oxygen level, pH, and other factors, tumor cells have to adjust their metabolic pathways to their microenvironment to sustain tumor development (Unterlass and Curtin, 2019; Liu R. et al., 2014; Stupnikova et al., 2025). Aberrant tumor metabolism supports the growth and proliferation of cancer cells and stimulates disease progression by satisfying increased demands for energy and biomass (Hanahan and Weinberg, 2011; Xia et al., 2022).

Discovered a century ago, aerobic glycolysis, the so-called Warburg effect, is one of the adaptive mechanisms involved in cancer cell metabolism (Warburg and Minami, 1923). Otto Warburg found that even in the sufficient presence of oxygen, cancer cells prefer to perform glycolysis by converting pyruvate to lactate rather than producing energy through oxidative phosphorylation (OXPHOS) as seen in normal tissues (Warburg, 1956; Wa and rburg, 1956). Despite the considerable advantage of OXPHOS over aerobic glycolysis in ATP production, aerobic glycolysis becomes relatively more beneficial for tumor tissue due to the higher speed of the glycolysis. By implementing rapid glucose conversion to lactate, tumor cells can produce ATP at a comparable rate as utilizing the complete oxidation of glucose to carbon dioxide. This provides an advantage in the competition for such a limited energy resource as glucose over surrounding normal cells (Liberti and Locasale, 2016).

Subsequently, enhanced glycolysis contributes to the accumulation of intermediate glycolytic products, which can be utilized to produce nucleotides, amino acids, and lipids necessary for tumor cell proliferation and biomass growth (Lunt and Vander Heiden, 2011). In addition, increased lactate secretion resulting from the Warburg effect decreases the pH of the tumor microenvironment, which may further enhance tumor invasiveness and reprogram surrounding cells into a pro-tumor phenotype (Estrella et al., 2013; Colegio et al., 2014). Alongside glucose metabolism reprogramming, cancer cells are characterized by alterations in lipids and amino acids metabolism, as well as other bioenergetic metabolic pathways. As a result, cancer cells establish an optimal metabolic pathway configuration for their survival, adaptation to stress and adverse conditions, and proliferation (Pavlova and Thompson, 2016). Altered metabolism also affects the tumor microenvironment, thereby helping PLC cells evade the immune response (Zhang et al., 2018; Lin et al., 2024). Thus, the detailed investigation of shared and unique metabolic reprogramming features is essential in immunometabolic studies of PLCs to develop a framework for modulating immune metabolism across the full spectrum of PLC subtypes (Zhou et al., 2026; Xue et al., 2025).

## Glucose metabolism

**FIGURE 1:**
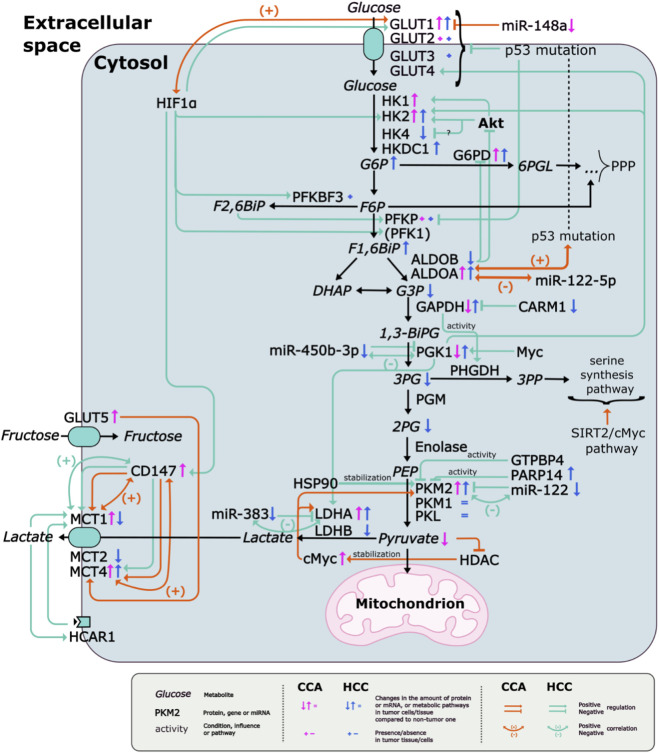
Metabolic reprogramming of glycolysis in cholangiocarcinoma and hepatocellular carcinoma.

Glycolysis is the central pathway of energy metabolism that leads to the formation of pyruvate, which can then be oxidized to lactate or transported into the mitochondria for entry into the tricarboxylic acid cycle (TCA) followed by OXPHOS. Glycolysis provides the cell with energy and intermediate compounds that can be directed into the pentose phosphate pathway (PPP), the pathway of hexosamine, or serine synthesis to produce essential cellular components such as amino acids and nucleotides (Park et al., 2020). Thus, tumor cells alter glucose metabolism towards enhanced glycolysis and increased lactate production to achieve the necessary energy level, maintain redox balance, and generate sufficient “building blocks” for proliferation (Liberti and Locasale, 2016; White and Schwab, 2015). The general changes in glucose metabolism are summarized in Figure 1.

### Glucose-6-phosphate is a cornerstone to biomass synthesis

#### Glucose uptake

The preceding step to glycolysis is glucose uptake by the cell, which is carried out by transporter proteins primarily of the glucose transporters (GLUT) family. In humans, 14 GLUT proteins are expressed, 11 of which are confirmed as capable of glucose transport. The most studied glucose transporters are GLUT1-4 (Mueckler and Thorens, 2013). Generally, different members of the GLUT family predominate in various organs and tissues. For instance, GLUT1 plays a crucial role in glucose uptake by brain cells, being the primary isoform of GLUT in astrocytes and brain endothelial cells, while GLUT2 is the main glucose transporter in hepatocytes; GLUT3 is essential for neurons in the brain, and GLUT4 is most prominently expressed in adipocytes (Mueckler and Thorens, 2013). In the liver, GLUT1 expression is observed exclusively in hepatocytes adjacent to the hepatic vein, whereas GLUT2 is expressed in all hepatocytes (Lazaridis et al., 1997). Additionally, GLUT1 expression has been demonstrated in the epithelial cells of bile ducts in rat liver (Lazaridis et al., 1997). However, the expression of glucose transporters in tissues significantly changes with the onset and progression of oncological diseases and may serve as a marker for pathological processes. GLUT1 overexpression detected in intrahepatic cholangiocarcinoma (iCCA) tissue is associated with poorer clinical outcomes, including reduced disease-related, overall, and disease-free survival, and correlates with increased tumor cell proliferation, migration, and invasiveness; conversely, GLUT1 inhibition (e.g., via siRNA) reduces migratory and invasive potential, supporting its role in tumor aggressiveness (Kubo et al., 2014; Tiemin et al., 2020). In addition, the correlation between GLUT1 expression and Hypoxia-inducible factor 1-alpha (HIF-1α) expression, whose level is also increased in tumor tissues from CCA patients, was also found. Silencing HIF-1α results in decreased proliferation, migration, and invasion of CCA cells, while HIF-1α overexpression leads to elevated rates of proliferation, migration, invasion, and cell cycle progression (Yu et al., 2020).

Ikeno et al. (2018) also demonstrated that patients with mass-forming iCCA and a high level of GLUT1 expression who underwent surgical resection had significantly lower survival rates than patients with low GLUT1 expression. Furthermore, GLUT1 expression is markedly lower in wild-type KRAS tumors compared to mutated ones, which aligns with the observation of shorter survival in patients with KRAS mutation (Ikeno et al., 2018). In a recent study investigating the connection between GLUT1 and the onset and progression of liver fluke-associated CCA, it was found that GLUT1 is highly expressed in CCA tissues but not in normal adjacent tissues. High GLUT1 expression was significantly associated with poor patient survival, and GLUT1 was identified as an independent predictor for poor prognosis. It was also found that the level of GLUT1 expression increases gradually during cholangiocarcinogenesis, while silencing GLUT1 significantly suppresses migration, growth, and invasion of CCA cells, which may indicate the important role of GLUT1 in the development and progression of liver fluke-associated CCA in humans (Thamrongwaranggoon et al., 2021). Additionally, GLUT1 was found to be a direct target of miR-148a, which is conversely decreased in iCCA tissue compared to adjacent non-tumor liver tissues. Consequently, the downregulation of miR-148a in CCA tissues may partially explain the overexpression of GLUT1 and its associated tumor progression and resistance (Tiemin et al., 2020). In the case of extrahepatic bile duct cancer (EHD), GLUT1 expression was detected in 76.9% of patients, which also significantly correlated with glucose metabolism as measured by [18F]-2-fluoro-2-deoxy-d-glucose (18F-FDG) uptake (Yoon et al., 2015).

Similarly to CCA, HCC depends on glucose flow, which is reflected in the expression of its transporters. Amann et al. found that, the level of GLUT1 mRNA in HCC tissues and cell lines is significantly higher than in primary human hepatocytes and matched non-tumor tissues (Amann et al., 2009). The elevated expression of GLUT1 directly correlates with proliferation level and invasiveness of HCC. At the same time, suppression of GLUT1 expression by siRNA weakens tumor growth and migratory potential, while inhibition of the transporter reduces glucose uptake and lactate secretion. It has also been shown that hypoxic conditions induce GLUT1 expression *in vitro*, which depends on the activation of HIF-1α (Amann et al., 2009). A similar conclusion was drawn in the study of HCC by Li et al. (2017b), which states GLUT1 as the downstream target of HIF-1α. Sun et al. (2016) confirmed significantly higher GLUT1 expression in HCC tissues compared with adjacent non-tumor tissues. Moreover, univariate analysis indicated that high GLUT1 expression is an unfavorable predictor of overall survival (OS) and is associated with shorter recurrence-free survival (RFS). Apart from GLUT1, Kim et al. (2017) show that SLC2A2 (GLUT2) expression in HCC is the highest compared to other members of this transporter family and is negatively associated with the late clinical stage of the disease. It was also shown that GLUT2 expression is positively associated with OS and may serve as a prognostic factor in HCC (Kim et al., 2017). In contrast, survival rates between GLUT2-positive and GLUT2-negative iCCA patients did not differ significantly; however, the GLUT2-positive iCCA group, compared with the GLUT2-negative iCCA group, exhibited statistically significant perihilar location and a non-mass forming type of tumor (Kubo et al., 2014). Interestingly, in a study performed by Paudyal et al. (2008a), GLUT2 expression in HCC significantly correlated with 18F-FDG uptake and reduced OS. Unlike Kim’s study, in which the majority of patients had early stage cancer, patients in Paudyal’s study who were positive for GLUT2 expression had late stage cancer with glycolysis entirely boosted by other enzymes and transporters. The role of GLUT3 has also been investigated in one of the recent studies focused on HCC development. Gao et al. (2019) demonstrated a significant association between a high level of GLUT3 expression in HCC samples and large tumor size, elevated α-fetoprotein level, poor histological differentiation of the tumor, and Tumor-Node-Metastasis (TNM) stages 3 and 4. Furthermore, high GLUT3 expression was also associated with reduced OS in patients.

#### Summary: glucose uptake alters similarly in both PLC

The expression of glucose transporters changes significantly with the occurrence of both CCA and HCC, which originates from the elevated needs in glucose consumption that is characteristic of cancer cells. In the case of CCA, a noticeable GLUT1 overexpression is observed, while GLUT2 expression occurs occasionally and is primarily specific to perihilar localisation. HCC, in turn, shows a more complicated picture. Based on the studies of Amann et al. (2009), we assume that the expression of all glucose transporters GLUT1-4 increases in HCC cells, while overexpression of GLUT1 is most pronounced. However, the aggressiveness of the tumor depends not only on an increase in the expression of a particular glucose transporter, but also on the ratio of their expressions. So, if an increase in expression is noted primarily in the GLUT2 transporter, then patients are likely to have better survival and a less aggressive course of liver cancer. Conversely, if GLUT1 and GLUT3, but not GLUT2, are predominantly overexpressed then there is a high probability of a more aggressive type of cancer. We explain it through the action of GLUT2 as a low-affinity, bidirectional transporter responsible for maintaining physiological glucose equilibration between the cytoplasm and the extracellular space (Guillam et al., 1998; Thorens, 2015). Prominent GLUT2 expression potentially prevents the intracellular accumulation of glucose-6-phosphate and the subsequent permanent activation of pro-glycolytic and lipogenic pathways. An alternative pathway may also be responsible for the release of glucose from the cell, which has not yet been established. Such a pathway is likely to be associated with sodium glucose transporters (SGLTs) belonging to the mammalian solute carrier family SLC5. However, SGLTs expression does not change in HCC (Cao et al., 2021), from which it can be inferred that the regulation of glucose levels in HCC cells occurs specifically through transporters of the GLUT family.

#### Glucose phosphorylation

Hexokinase (HK) is the first regulatory enzyme in glucose metabolism, phosphorylating glucose in the cell cytoplasm to glucose-6-phosphate (G6P). Currently, five types of hexokinases are known: HK1, HK2, HK3, HK4, and HKDC1, each with its own tissue specificity and function (Farooq et al., 2023). Yu et al. found that the expression of HK1 protein in hilar cholangiocarcinoma (hCCA) tissue is markedly increased compared to normal tissue samples. Moreover, high HK1 level correlates with poor OS and DFS, and is also significantly associated with lymph node metastasis and stages 3 and 4 of the disease. However, HK1 is not an independent prognostic factor for hCCA (Yu et al., 2015). In the cases of opisthorchis viverrini-associated CCA, HK2 is distinctly expressed in cancer-affected bile ducts, whereas it is rarely found in the epithelium of normal bile ducts. At the same time, suppression of HK2 using siRNA significantly reduces migration, proliferation, and invasion of CCA cell lines, which may indicate a crucial role for HK2 in cholangiocarcinogenesis and suggest a potential of HK2 as a therapeutic target for cancer treatment (Thamrongwaranggoon et al., 2017). HK2 expression has also been observed in EHD, not associated with specific risk factors. HK2 is expressed in 84.6% of cases, but does not correlate with glucose flow or GLUT1 expression (Yoon et al., 2015). Interestingly, 7 years prior to this study, a significant correlation was identified between HK2 and 18F-FDG uptake, as well as an association between HK2 and GLUT1 in CCA tissues (Paudyal et al., 2008b). This discrepancy could be explained by small sample sizes, tissue heterogeneity, and differences in localization and stage, underscoring the necessity to consider disease heterogeneity and the corresponding samples during research.

HCC cells also acquire some specific features in the expression and regulation of hexokinases. HK4, a low-glucose-affinity hexokinase dominant in normally differentiated hepatocytes, is replaced with HK2, which is characterized by a higher affinity for glucose (Farooq et al., 2023; DeWaal et al., 2018). During HCC carcinogenesis—from normal liver tissue through non-dysplastic cirrhosis (NDC) to liver cell dysplasia (LCD) in cirrhosis, and finally to HCC—the level of HK2 is gradually increasing, correlating with more aggressive histological features of HCC (Guzman et al., 2015). The significance of mitochondrial binding HK2 was demonstrated in the study by DeWaal et al. (2018), in which silencing this enzyme in human HCC cells led to increased cell death and inhibition of tumorigenesis. Notably, neither HK4 expression nor expression of a mutant HK2 with mitochondrial binding deficiency helps to restore oncogenesis in HK2 knockdown cell lines, which may partially explain the preference for the transition from HK4 to HK2 in cancer cells. In experiments by Gwak et al. (2005) on human HCC cell lines, hypoxic conditions were shown to induce HK2 expression through the HIF-1α-dependent mechanism, thereby stimulating cancer cell growth. HK2 inhibition significantly suppresses HCC growth due to the induction of apoptosis. This result is consistent with earlier studies regarding the impact of hypoxia on HIF-1 stabilization, leading to an increase in HK2 expression followed by inhibition of apoptosis in HCC cells (Geschwind et al., 2004). It is important to note that the downstream targets of HIF-1α are specifically HK2 and GLUT1, rather than other glycolytic enzymes (Li et al., 2017b). In addition to HK2, changes in the expression of other hexokinases can also be observed. Zhang et al. found that HKDC1 expression is elevated in HCC tissue compared to adjacent tissues, and high expression of HKDC1 is associated with poor OS. At the same time, silencing HKDC1 suppresses proliferation and migration of HCC cells *in vitro*, which presumably can occur through inhibition of the Wnt/β-catenin signaling pathway. Thus, HKDC1 can also be considered as a new therapeutic target for HCC treatment (Zhang et al., 2016).

G6P, produced by HK phosphorylation of glucose, can either remain in the glycolytic pathway and subsequently serve as a substrate for the production of fructose-6-phosphate (F6P), or it can participate in PPP (Park et al., 2020). The PPP is one of the central players in cellular biosynthetic metabolism, maintaining carbon homeostasis and serving as a source of precursors for nucleotide and amino acid synthesis. Last but not least, this metabolic pathway provides cells with NADPH, reducing equivalents essential for fatty acids (FA) *de novo* synthesis, maintaining redox balance, and combating oxidative stress (Stincone et al., 2015). The increased expression and activity of glucose-6-phosphate dehydrogenase (G6PD), the rate-limiting enzyme of the PPP, is observed in HCC tumor tissue compared to healthy liver tissue and paracarcinoma tissue (Kowalik et al., 2017). Similarly, in CCA, an increase in G6PD expression has been noted in tumor tissue compared to non-tumor tissue (Zheng et al., 2023).

#### Summary: glucose phosphorylation changes similarly in both PLC

The increase in the expression of hexokinases, especially HK2, the high-affinity isoform, is an expected response to an increased G6P demand to support PPP. Stimulation of the PPP, on the one hand, provides the cell with components for biomass synthesis. On the other hand, it slows down glycolysis due to the diversion of G6P to PPP, reducing the rate of ATP and pyruvate synthesis. Thus, enhancing hexokinase activity is essential for the cell to maintain a sufficient glycolysis rate.

### Downstream decomposition of G6P

#### Fructokinases

Phosphofructokinase 1 (PFK1) is a regulatory and rate-limiting enzyme in glycolysis, responsible for the phosphorylation of F6P to fructose-1,6-bisphosphate (F1,6BP). PFK1 has three isoforms: muscle type (PFKM), liver type (PFKL), and platelet type (PFKP), which are distributed in the body in different proportions depending on the type of tissue (Dunaway et al., 1988). This enzyme is allosterically activated by fructose-2,6-bisphosphate (F2,6BP), which is produced from F6P by the enzyme 6-phosphofructo-2-kinase/fructose-2,6-bisphosphatase (PFK2/F2,6BPase or PFKFB). PFKFB has four isoforms (PFKFB1-4), each with its specific kinase and phosphatase activity (Hay, 2016; Lee et al., 2017). Fujiwara et al. (2019) discovered the expression of PFKP protein in 69 out of 101 patients (68%) with iCCA, and expression levels were significantly higher in CCA cases carrying IDH mutations. Thus, the enhancement of glycolysis caused by IDH1 mutation can occur through the upregulation of the PFKP gene. In the study by Yu et al. (2015), a high level of PFKFB expression was identified in 56% of cases of hCCA; however, no correlation was established between the expression of this enzyme and tumor or healthy tissues. Unlike CCA, in the case of HCC, several research groups have managed to establish the significance of PFKFB. For example, it was shown that PFKFB3 expression is strongly associated with resistance to sorafenib, one of the most effective drugs for treating HCC (Li et al., 2017c). Furthermore, high expression of PFKFB3 is associated with poorer patient survival outcomes and large tumor size. Apart from the direct involvement of PFKFB3 in glycolysis, the enzyme also promotes HCC growth through the PFKFB3/AKT/ERCC1 signaling pathway due to its localization mainly in the nucleus (Shi et al., 2018). Additionally, it was shown that metformin reduces the expression levels of PFKFB3 and PFK1 by suppressing HIF-1α, thereby decreasing glycolytic flux and significantly disrupting hepatoma cell proliferation (Hu et al., 2019).

#### Aldolases

The subsequent glycolytic enzyme, Aldolase A (ALDOA), which is responsible for the cleavage of F1,6BP to D-glyceraldehyde 3-phosphate (G3P) and dihydroxyacetone phosphate (DHAP) is significantly overexpressed in iCCA tumor tissue at both the mRNA and protein levels compared to adjacent paracarcinoma tissues. Furthermore, the high expression level of ALDOA correlates with tumor malignancy, metastasis, and poor prognosis in CCA patients. Knockdown of ALDOA in iCCA cells leads to glycolysis inhibition, decreased levels of G3P, DHAP, and 3-phosphoglycerate (3PG), decreased proliferation, migration, and invasion *in vitro*, as well as inhibition of tumor growth *in vivo*. The enzymatic activity of ALDOA plays a crucial role in CCA progression, affecting proliferation and invasion levels of CCA cells even in the case of unchanged protein expression (Li et al., 2021). ALDOA knockdown also promotes apoptosis, while the reduction of apoptosis induced by ALDOA overexpression in CCA cells can be reversed by miR-122-5p, the level of which is decreased in bile duct carcinoma tumors and negatively correlates with ALDOA expression. By influencing proliferation, invasion, and growth of CCA cells, the ALDOA/miR-122-5p axis may play a significant biological role in the development of CCA (Xu et al., 2018).

In HCC, mRNA and protein expression levels of ALDOA are also significantly elevated in tumor tissue compared to normal non-tumor tissue, which is particularly characteristic for P53-mutated tumors (Tang et al., 2021; Castaldo et al., 2000). Meanwhile, the potential mechanism of increased ALDOA expression in HCC may involve an increase in the DNA copy number and methylation (Tang et al., 2021). Moreover, ALDOA overexpression significantly correlates with clinicopathologic characteristics of HCC patients, such as TNM stage, histological grade, T stage, and living status. Thus, ALDOA expression, which negatively correlates with OS and DFS, serves as an independent prognostic risk factor for HCC (Tang et al., 2021). Gene Set Enrichment Analysis (GSEA) revealed that ALDOA activation is associated with glucose catabolism, the cell cycle, DNA replication, and AKT/mTOR and MYC signaling pathways in HCC; however, further studies are required to clarify the underlying mechanisms (Tang et al., 2021). In murine and human hepatocellular carcinoma cell lines, it was found that disruption of ALDOA catalytic activity in a glucose-enriched environment leads to the accumulation of F1,6BP, severe energy stress (decreased ATP and Pi levels), and cell cycle arrest (Snaebjornsson et al., 2025). Interestingly, ALDOA may contribute to HCC development not only by performing its canonical function as a bidirectional glycolytic enzyme but also by accelerating mRNA translation and enhancing overall protein biosynthesis in cells, thereby promoting cancer growth and metastasis (Song et al., 2023).

In contrast to ALDOA, which is normally repressed in liver tissues (Lebherz and Rutter, 1969), the major Aldolase isoform in healthy adult liver, Aldolase B (ALDOB) (Gregori et al., 2002). In HCC tissue, ALDOB mRNA expression is often markedly reduced or even absent. Moreover, downregulation of ALDOB is significantly associated with high tumor grade, portal vein invasion, early tumor recurrence (ETR), and a lower 5-year survival. Notably, at early stages of HCC (e.g., stage II), downregulation of ALDOB can serve as a predictor of frequent ETR and poor prognosis (Peng et al., 2008). ALDOB can directly bind to G6PD, an enzyme that limits PPP rate, thereby inhibiting G6PD activity and suppressing cell proliferation. This effect is enhanced within the ALDOB-G6PD-p53 complex, in which ALDOB performs a stabilizing function, thereby enhancing p53-mediated inhibition of G6PD in HCC cells and reducing oxidative metabolism in PPP regardless of the enzymatic activity of ALDOB. Conversely, loss of ALDOB or disruption of the ALDOB-G6PD interaction leads to destabilization of the complex, which weakens the inhibitory effect of p53 and ALDOB on G6PD and reinvigorating PPP metabolism, which is crucial for tumor growth (Li et al., 2020). Another non-enzymatic tumor-suppressive function of ALDOB in HCC development is the direct interaction of ALDOB with Akt. It inhibits Akt phosphorylation and kinase activity, thereby attenuating downstream signaling independently of ALDOB enzymatic activity. Interestingly, it was also shown increased expression of HK1 and HK2 and decreased expression of HK4, which are downstream targets of Akt, in mice with ALDOB knockout. This finding aligns with the switch from HK4 to HK2, which promotes enhanced glucose metabolism during HCC development (He et al., 2020). Beyond its effects on glucose uptake, ALDOB-mediated suppression of Akt kinase activity may also lead to a reduction in cellular metabolism in glycolysis and tricarboxylic acid (TCA) cycle, and inhibition of cell cycle progression, directly contributing to the suppression of cell growth and oncogenesis. The ALDOB-mediated suppression of Akt activity and the associated reduction in tumor growth may occur via the ALDOB-Akt-PP2A complex. In this complex, ALDOB interacts with phosphorylated Akt, recruiting protein phosphatase 2A (PP2A), which subsequently dephosphorylates Akt, thereby reducing Akt’s activity. Loss of ALDOB or disruption of the ALDOB-Akt interaction restores Akt activity previously suppressed by the complex, leading to corresponding oncogenic consequences (He et al., 2020). Thus, Li et al. (2020) proposed two mechanisms by which ALDOB affects HCC progression: through physical interactions involving Aldob-G6PD and ALDOB-Akt. Notably, decreased ALDOB expression, combined with increased G6PD activity or upregulation of phosphorylated Akt (p-Akt), serve as predictors of poor prognosis in patients with HCC (Li et al., 2020; He et al., 2020).

#### Triose section

A peculiar regulation of glycolysis in CCA cells occurs at the level of glyceraldehyde-3-phosphate dehydrogenase (GAPDH) and phosphoglycerate kinase 1 (PGK1), whose expression, as demonstrated by Guo et al., is significantly reduced in large-duct type CCA tissues. Knockdown of these enzymes in CCA cell lines enhances cellular proliferation (Guo et al., 2022). Thus, an atypical suppression of glycolysis is observed in iCCA, potentially serving as a distinctive feature of the large-duct-type. In HCC, by contrast, both protein and mRNA expression levels of PGK1 are significantly elevated, with a progressive increase of the expression observed from normal liver, through cirrhosis, to HCC. Moreover, HCC patients with high PGK1 expression display significantly poorer survival and worse malignancy compared to PGK1-negative patients (Hu et al., 2017; Xie et al., 2017; Daskalow et al., 2009; Yi et al., 2024). Chen et al. (2019) further confirmed the upregulation of PGK1 protein and mRNA expression in HCC and identified miR-450b-3p as one of the possible PGK1 regulators. The expression level of miR-450b-3p is significantly downregulated in HCC tissues compared to adjacent non-tumor tissues and inversely correlates with PGK1 protein, but not mRNA, levels. At the same time, PGK1 was identified as a direct target of miR-450b-3p, and PGK1 overexpression can reverse the inhibitory effects of miR-450b-3p on HCC cell proliferation and division, underscoring the oncogenic role of PGK1 in hepatocarcinogenesis (Chen et al., 2019). Unlike miR-450b-3p, which inhibits PGK1 expression, MYC induces PGK1, increasing the expression of key glycolytic transporters and enzymes, such as GLUT4, HK2, and lactate dehydrogenase A (LDHA). Consequently, glucose uptake, glycolytic rate, lactate production, and ATP levels rise in HCC. Thus, MYC-dependent expression of PGK-1 provides a significant boost for the maintenance of HCC viability, metastasis, and tumor growth (Xie et al., 2017). MYC-dependent regulation of PGK1 was also noted in an earlier study, in which c-Myc knockdown, but not HIF-1α, resulted in a sharp decrease in PGK1 expression in HCC cell lines. Additionally, acetylation of PGK1 at K323 site, regulated positively by PCAF and negatively by SIRT7, enhances PGK1 enzymatic activity, promoting cellular proliferation and oncogenesis in HCC (Hu et al., 2017). As in the case of PGK1, GAPDH mRNA expression is significantly increased in HCC tumor tissues compared to adjacent non-tumor and healthy liver tissues (Gong et al., 1996). Furthermore, Liu et al. demonstrated that GAPDH contributes to HCC development by enhancing the activity of phosphoglycerate dehydrogenase (PHGDH), a rate-limiting enzyme in serine biosynthesis. This redirection of glycolytic flux toward biomass synthesis supports tumor growth (Liu et al., 2017). One of the possible regulators of GAPDH in HCC is coactivator-associated arginine methyltransferase 1 (CARM1). By methylating GAPDH at the R234 site, CARM1 reduces the catalytic activity of GAPDH, suppressing glycolysis and delaying HCC cell proliferation *in vitro* and *in vivo*. Furthermore, GAPDH receives glucose availability signals from CARM1, whose protein level is hypothesized to be regulated by AMPK-mediated mechanisms. Thus, GAPDH methylation plays a pivotal role in cancer cell metabolic regulation and nutrient availability response. Notably, in HCC patients, CARM1 protein level and R234 methylation are lower in tumor tissues compared to surrounding normal tissues, with a positive correlation between CARM1 level and R234 methylation (Zhong et al., 2018).

A concomitant effect of altered glucose transporters and key glycolytic enzymes expression is the change in levels of various glycolytic metabolites. HCC tissues exhibit a significant increase in levels of G6P and F1,6BP compared to non-tumor tissues (Huang et al., 2013), which can be explained by the replacement of the low-affinity glucose hexokinase HK4 with the high-affinity HK2 during hepatocarcinogenesis, accompanied by overall HK2 upregulation that stimulates a faster conversion of glucose to Glucose-6P, thereby accelerating glycolysis (Farooq et al., 2023; DeWaal et al., 2018). Interestingly, the downstream metabolite G3P level is significantly reduced in HCC tissues (Huang et al., 2013) due to probably a substantial increase in the expression of PGK1 (Xie et al., 2017) and GAPDH (Gong et al., 1996), which leads to the depletion of G3P levels in HCC tissues, even with increased expression of ALDOA (Tang et al., 2021; Castaldo et al., 2000). In addition to G3P, HCC exhibit approximately a twofold depletion of downstream metabolites G3P and Glycerol-2-phosphate (G2P) in tumor tissues compared to control (Beyoğlu et al., 2013). Logically, this can be explained by the utilization of these intermediates for the synthesis of glycerol, as well as serine, which is then converted into other amino acids. However, further studies are needed to confirm this hypothesis.

#### Pyruvate formation

The final shared enzyme both for aerobic glycolysis and OXPHOS is pyruvate kinase (PK), which catalyzes the conversion of phosphoenolpyruvate to pyruvate. In mammals, PK exists in four isoforms—PKL, PKR, PKM1, and PKM2 (Imamura and Tanaka, 1982). PKL and PKR are produced from a single gene using different promoters, but PKL is primarily found in hepatic cells, while PKR is predominantly expressed in erythrocytes (Noguchi et al., 1987; Israelsen and Vander Heiden, 2015). PKM1 and PKM2 arise from another gene through alternative RNA splicing (Noguchi et al., 1986). PKM1 is present in muscle, heart, and brain tissues, and PKM2, primarily embryonic isoform, is highly expressed in proliferative and cancerous cells (Israelsen and Vander Heiden, 2015). PKM2 can exist in four states with varying enzymatic activity, particularly a low-activity dimeric form and a highly active tetrameric form. Based on this, PKM2 can be involved in metabolic reprogramming of cancer cells, promoting the accumulation of glycolytic intermediates as building blocks for tumor development (Alquraishi et al., 2019). PKM2 is highly expressed in iCCA tissues, and patients with a lower expression level of this enzyme have significantly longer median survival time after surgery. PKM2 expression in CCA is significantly associated with lymph nodes and distant metastases. Targeted knockdown of the enzyme leads to inhibition of tumor growth, invasion, and migration (Qian et al., 2020). The association of PKM2 expression and lymph node metastasis was also found in an earlier study, where PKM2-positive tumors were observed in 85.8% of CCA patients with lymph node metastasis (Suzuki et al., 2015). PKM2 expression was also found to be upregulated in hCCA tissue and associated with lymph node metastasis, neural invasion, and poor or lack of tumor differentiation. hCCA patients with a high level of PKM2 expression in tumor tissue demonstrate significantly shorter DFS and OS (Yu et al., 2015). PKM2 expression has been identified both in the cytoplasm of hCCA cells, which is characteristic of a glycolytic enzyme, and in the nuclei of poorly differentiated and undifferentiated hCCA cells, as well as in nuclei of cells at the invasion margins of primary tumors and metastatic lesions (Yu et al., 2015). Nuclear localization, which, among other things, is characteristic of the almost inactive dimeric form of PKM2 (Alquraishi et al., 2019), indicates an extensive role of PKM2 in cholangiocarcinoma carcinogenesis through gene expression regulation.

The expression of PKL, the predominant pyruvate kinase in a healthy liver, remains unchanged during hepatocarcinogenesis, and the same is true for the PKM1 isoform. However, PKM2 is often overexpressed in human HCCs at both the mRNA and protein levels (in 48.3% and 68.8% of cases, respectively), and associated with shorter OS of HCC patients, aggressiveness of the disease, and a higher recurrence rate (Wong et al., 2014). PKM2 knockdown *in vitro* suppresses aerobic glycolysis, reflected in a decrease in lactate accumulation, glucose uptake rate and acceleration of oxidative stress in cells. Further, knockdown *in vivo* suppresses tumor growth and metastasis. The mechanism of switching from PKL to PKM2 isoforms during HCC development relies on miR-122, one of the most abundantly expressed miRNAs in the human liver, but downregulated in HCC. miR-122 acts as a suppressor of PKM2 (but not PKL), reducing its expression but not the activity. Moreover, the expression of miR-122 and PKM2 have an inverse correlation in human HCC and non-tumorous liver tissues (Wong et al., 2014). Combining the results with earlier work led by Liu A. M. et al. (2014), we note the important role of the miR-122/PKM2 axis in the development of HCC. Unlike miR-122, which is a direct suppressor of PKM2 expression, anti-apoptotic protein poly(ADP-ribose) polymerase 14 (PARP14) regulates its activity. PARP14 is upregulated in HCC compared with their adjacent non-tumor tissues and reduces PKM2 activity, thereby contributing to a reduced glycolytic rate and accumulation of intermediates for tumor development. This regulation occurs along the PARP14-JNK1-PKM2 axis, where PARP14 inactivates the pro-apoptotic kinase JNK1, which in turn inhibits the JNK1-dependent phosphorylation of PKM2 at Thr-365, thereby preventing the PKM2 enzyme from becoming more highly active (Iansant et al., 2015). Phosphorylation at other sites can have a completely opposite effect: Thr-328 phosphorylation, induced by heat shock protein 90 (HSP90) and mediated by protein kinase glycogen synthase kinase-3b (GSK-3b), on the contrary, increases the stability of PKM2, thereby contributing to its abundance in HCC cells. In addition, the positive expression of PKM2 and HSP90, and especially their combination, predicts the poor prognosis of HCC patients (Xu et al., 2017). Increased aerobic glycolysis in HCC cells can also occur through PKM2 sumoylation. Guanosine triphosphate binding protein 4 (GTPBP4) induces the formation of the PKM2 dimer, a less active form of hexokinase, through sumoylation via the UBA2-SUMO1 axis, thereby enhancing the Warburg effect. Moreover, GTPBP4-induced PKM2 sumoylation triggers dimer translocations from the cytosol to the nucleus, which can activate the STAT3 signaling pathway and the epithelial-mesenchymal transition (EMT), thereby contributing to the progression and metastasis of HCC (Zhou et al., 2022). Nuclear PKM2 also affects HCC cell proliferation and apoptosis by regulating HIF-1α and Bcl-xL expression. Dong et al. showed that overexpression of PKM2, characteristic of HCC tissues compared with paracarcinoma tissues, enhances the proliferation of HCC cells by increasing the expression of HIF-1α and Bcl-xL (Dong et al., 2015). HIF-1α is known to stimulate the proliferation of hepatoma cells and increase their resistance to apoptosis through regulation of Forkhead box M1 (FoxM1) expression (Xia et al., 2012), while high Bcl-xL expression has an antiapoptotic role and significantly correlates with portal cancer invasion and poor prognosis of HCC patients (Watanabe et al., 2004). Moreover, the nuclear expression of PKM2 itself can serve as an independent risk factor for early recurrence after radical tumor resection in patients with HCC (Fan et al., 2016). Another independent and significant risk factor for early recurrence and decreased OS was the combination of PKM2 and TRIM35 expressions in patients with HCC. At the same time, patients with PCM2(+) and TRIM35(−) expressions had a significantly worse prognosis (Chen Z. et al., 2015).

#### Summary: glycolysis undergoes convergent alterations despite the type of PLC

Glycolytic enzymes play a crucial role in glucose metabolism reprogramming, regulation of aerobic glycolysis, serine and amino acids synthesis, and thus the development and progression of CCA and HCC. Despite the minor discrepancies between glucose uptake and metabolism in HCC and CCA, which reflect altered tumor environment and glucose availability, overall metabolic alterations are similar for both PLCs.

### The ambiguous role of lactate

#### Aerobic glycolysis

Lactate dehydrogenase A is an essential enzyme in cancer development that converts pyruvate into lactate. By promoting the production of lactate, LDHA lowers the pH to enhance the ability of cancer to invade, replenishes the NAD^+^ pool for glycolysis, directly inhibits apoptosis, and mediates the escape of the tumor from the immune response (Feng et al., 2018). In CCA, LDHA is overexpressed in tumor tissue, while the enzyme downregulation leads to a decrease in cell growth and induction of apoptosis. Interestingly, LDHA inhibition also promotes an increase in cytoplasmic reactive oxygen species (ROS) levels, which may indicate a link between overexpression of the enzyme in CCA and ROS downregulation (Yu et al., 2014). High LDHA level is associated with shorter survival of CCA patients, and the enzyme itself is an independent prognostic risk factor (Thonsri et al., 2017). Increased lactate production, the well-known result of the Warburg effect, leads to the accumulation of lactate in the tumor tissue, which, in turn, paradoxically, reverses the phenotype of cancer cells back from aerobic glycolysis to OXPHOS (Wu et al., 2016). Long-term (over 2 weeks) exposure of CCA cells to lactic acidosis (LLA) conditions leads to an increase in intracellular lactate, a decrease in intracellular pH, but more importantly, an enhanced capacity for cell migration and a phenotypic switch from glycolysis to mitochondrial respiration with an increase in mitochondrial mass. The transcriptomic analysis also revealed the upregulation of genes associated with migration and epithelial-mesenchymal transition in LLA conditions, particularly thrombospondin 1 (THBS1). The THBS1 protein is significantly elevated in human CCA tissues, and its high expression is associated with an unfavorable patient outcome. Moreover, patients with simultaneous high expression of THBS1 and LDHA had the shortest survival. Interestingly, inhibition of THBS1 and protein neutralization by antibodies in CCA cells reversed LLA-induced metabolic reprogramming of cells, canceling the increase in oxygen consumption rate and mitochondrial mass, as well as decreasing cellular motility (Thamrongwaranggoon et al., 2023). Zhang et al. (2019) discovered a rather curious feedback loop involving LDHA, which contributes to the Warburg effect and cell proliferation. The increased level of cMYC in CCA patients tissues promotes LDHA and PKM2 levels, thereby reducing the amount of intracellular pyruvate. A low level of pyruvate results in decreased inhibition of HDAC3, the main target of pyruvate, thereby protecting cells from apoptosis (Zhang et al., 2019; Yin et al., 2017). In addition, HDAC3 itself is a stabilizer of the cMYC protein by preferential deacetylation of cMyc at the K323 site, which closes the feedback loop and promotes a low pyruvate level. By examining tissues from patients with CCA, this study also found that cMYC, PKM2, and LDHA levels are significantly elevated in tumor tissue and associated with poor prognosis, while pyruvate level is conversely decreased, further supporting the feedback loop hypothesis (Zhang et al., 2019).

Similarly, LDHA protein expression is also increased in HCC cells and associated with a high metastatic potential. Knockdown of this enzyme leads to a decrease in proliferation and induction of apoptosis of HCC cells *in vitro* and inhibition of metastasis *in vivo*, which emphasizes the important role of LDHA in the development and metastasis of HCC (Sheng et al., 2012). The level of LDHB, another isoform of LDH enzyme, on the contrary, is significantly reduced in the HCC tumor tissue compared to non-tumor tissue, becoming an independent prognostic factor. Patients with a lower level of LDHB expression have significantly lower OS and DFS. The enzyme is associated with aggressiveness, metastasis to lymph nodes, stage TNM, and degree of vascular invasion (Chen R. et al., 2015). The increased expression of miR-383, one of the LDHA expression regulators in liver cells, inhibits glycolysis and, thus, lactate production, LDHA-induced proliferation and invasion of HCC cells, thereby acting as a tumor suppressor. However, the expression level of miR-383 is significantly downregulated in HCC tissues and, as expected, inversely correlates with the level of LDHA expression. So, targeting the miR-383-LDHA axis can serve as a therapeutic strategy in the fight against HCC (Fang et al., 2017). A change in LDH expression in tumor tissue can lead to a change in the level of this protein in body fluids. Several groups of researchers have demonstrated that a high level of preoperative LDH in the serum and plasma of patients can serve as an independent prognostic indicator of the worst OS and DFS of HCC patients and also correlates with various clinical and pathological features (Zhang et al., 2015; Wang Z. X. et al., 2015; Su et al., 2023; Kong et al., 2018; Hu et al., 2015).

#### Lactate efflux and uptake

The development of LLA conditions within the tumor leads to the inhibition of glycolytic enzymes such as LDH (Stambaugh and Post, 1966), PFK1 (Trivedi and Danforth, 1966), followed by a decrease in the rate of glycolysis, loss of the ability to regenerate NAD+ and, ultimately, cell death. Therefore, to prevent this outcome, tumor cells must intensify lactate transport into the extracellular space via monocarboxylate transporters (MCTs) (Payen et al., 2020). MCT1-4 carry out bidirectional lactate transport across the plasma membrane, but expression of one or another form of MCT varies according to tissue type and cellular requirement. In normal tissues, MCT1 is expressed ubiquitously; MCT2 is restricted primarily to lactate-consuming cells: hepatocytes and brain neurons; MCT3 - in the choroid plexus epithelia of the eye and retinal pigment, MCT4 - in glycolytic cells as it has the lowest affinity to pyruvate ensuring its complete utilization in cell. At the same time, MCTs differ in their affinity for lactate (MCT2 has the highest affinity, followed by MCT1, MCT3, and finally, least affine but most selective, MCT4) (Payen et al., 2020).

In CCA tissues, the expression levels of MCT1 and MCT4 are increased compared to normal adjacent tissues of the bile ducts, and high expression of MCT4 is an independent prognostic factor for poor prognosis (Dana et al., 2020). In this case, CD147 can serve as a regulator of the expression of these transporters in CCA, the level of which is elevated in CCA tissues and directly correlates with the expression levels of MCT1 and MCT4. By activating the Akt-FOXO3-NFkB-MCT1/4 axis, CD147 increases the migration and invasion of CCA tumor cells, thereby contributing to the development of the disease (Dana et al., 2020). An increased level of MCT4 expression in human CCA tissues was also found in the work of Suwannakul et al., and it was observed that MCT4 expression is influenced by GLUT5, a high-affinity fructose transporter overexpressed in CCA tissues (Suwannakul et al., 2022).

MCTs expression behavior during hepatocarcinogenesis shows MCT2 downregulation during the transition from healthy tissue to HCC and, further, to metastatic samples. MCT4 expression, on the contrary, gradually increases at the same time. MCT1 is frequently (96% of cases) expressed in non-tumor tissue, while in HCC and metastases, the frequency of occurrence drops to 50%–60%. Interestingly, positive expression of MCT1, but not MCT4, in the plasma membrane of HCC cells is associated with CD147 upregulation. MCT4 expression is also associated with GLUT1, which is upregulated in HCC and its metastases (Alves et al., 2014). Thus, GLUT1 upregulation combined with high expression of glycolytic cells-associated MCT4, promotes the development of cancer cells through the accumulation of intermediate glycolysis metabolites, which are directed into PPP and the synthesis of other cell building materials. MCT4 upregulation in HCC cells is also associated with high serum alpha fetoprotein (AFP) level, large tumor size, late-stage disease, worse OS and DFS; thus becoming an independent prognostic factor after resection (Ohno et al., 2014). Interestingly, the expression level of this transporter is also associated with the tumor response of HCC patients to TACE treatment, where low MCT4 level is associated with a good response and OS, while high or medium levels are associated with a worse prognosis. MCT4 inhibition leads to a significant decrease in migration, proliferation and invasion, as well as a decrease in HIF-1α expression and Akt phosphorylation (Gao et al., 2015). Similarly, MCT1 inhibition leads to a decrease in lactate excretion, a decrease in Akt activity, inhibition of glucose consumption, and a reduction of HCC cells proliferation rate (Huang et al., 2014). In HCC cells, CD147 activates the PI3K/Akt/MDM2 pathway through MCT1-mediated lactate export, leading to the degradation of tumor suppressor p53. The presence of the exogenous p53 negatively affects the rate of glycolysis by inhibiting GLUT and PFK, enhancing the biogenesis and respiratory function of mitochondria, which prevents tumor development. Thus, MCT1 contributes additionally to maintaining HCC viability and growth (Huang et al., 2014).

MCTs also participate in mechanisms protecting from HCC cell death. Under hypoxic conditions, HIF-1α-mediated upregulation of CD147 protein expression occurs in HCC cells, leading to increased expression levels of MCT1 and MCT4. This results in enhanced lactate secretion and glycolysis rates, ultimately avoiding apoptosis that could be triggered by hypoxic conditions. Notably, there is a feedback loop in the MCT4-CD147 interaction: MCT4 expression is necessary for the membrane localization of CD147, which can also influence the level of cellular apoptosis. Indeed, in human hepatic stellate cells, in which MCT4 deficiency is observed under hypoxic conditions, the membrane localization of CD147 is disrupted, and the percentage of apoptosis increases under hypoxia (Ke et al., 2014). At the same time, lactate uptake via the MCT1 transporter also plays a crucial role in protecting HCC cells from death. At high extracellular lactate levels, as demonstrated by Zhao et al. (2020), liver cancer cells are resistant to ferroptosis—a programmed oxidative cell death caused by lipid peroxidation. The following mechanism of this resistance has been proposed: extracellular lactate stimulates the expression of MCT1 and hydroxycarboxylic acid receptor 1 (HCAR1), a lactate receptor that is also a positive regulator of MCT1 expression. This leads to increased lactate uptake, resulting in enhanced ATP production in liver tumor cells, followed by the deactivation of AMP-activated protein kinase (AMPK) due to a decrease in the AMP/ATP ratio. Consequently, the AMPK-mediated inhibition of sterol regulatory element-binding protein 1 (SREBP1) is removed, which in turn enhances the regulation of downstream stearoyl-coenzyme A (CoA) desaturase-1 (SCD1), which catalyze the biosynthesis of anti-ferroptosis monounsaturated fatty acids (MUFAs), and downregulates acyl-coenzyme A (CoA) synthetase (ACSL4), an enzyme for pro-ferroptosis polyunsaturated fatty acids (PUFAs) synthesis. As PUFAs are the preferred substrate for lipid peroxidation, the increased production of MUFAs and decreased levels of PUFAs can potentially reduce ROS accumulation in HCC cells, thereby enhancing their resistance to ferroptosis (Zhao et al., 2020).

#### Summary: lactate production and accumulation exhibit similar patterns across various types of PLC

Both CCA and HCC increase lactate production through an increased expression of LDHA and, typically, MCT4, a bidirectional lactate transporter with the lowest affinity for lactate. At the same time, a marked decrease in high-affinity lactate transporters MCT1 and MCT2 in the HCC tumor tissue indicates the benefits of lactate accumulation in HCC cancer cells. Conversely, an increase in MCT1 expression, which reduces the likelihood of LLA development, was observed in the case of CCA. Elevated extracellular lactate concentrations facilitate excessive lactate conversion into pyruvate in both CCA and HCC, enhancing tumor cell respiration. Accordingly, pyruvate deficiency caused by the increased consumption of G6P and other intermediate metabolites for biomass synthesis, the uptake of extracellular lactate released by other tumor cells in the tumor environment becomes one of the energy sources in the cell through the activation of OXPHOS. Thus, by regulating the expression of MCT1, MCT2, and MCT4, tumor cells maintain the lactate level according to current needs, but at the same time avoid excessive acidification. Moreover, with the bidirectional transport of lactate and its redistribution between cells with different availability of nutrients, it becomes possible to supply tumor cells evenly with energy and building blocks by converting lactate back into pyruvate.

Alterations in glycolysis and lactate metabolism enzymes are reflected in the levels of metabolites. An increased level of lactate and a decreased glucose level is observed in HCC patients’ tissues compared to non-tumor tissue samples (Sheng et al., 2012; Teilhet et al., 2017). However, it is important to note that information about metabolites’ intra- or extracellular localization is lost during their extraction. Consequently, the reduction in overall glucose level in HCC tissues may be attributed to the difficulty of nutrient delivery to the tumor microenvironment, which is characteristic of cancer (Cui et al., 2023), as well as to the rapid incorporation of intracellular glucose into glycolytic metabolism. In CCA, a decrease in intracellular pyruvate level in tumor cells (Zhang et al., 2019) stemmed from increased aerobic glycolysis, particularly the enhanced conversion of pyruvate to lactate driven by elevated LDHA expression (Yu et al., 2014). However, further studies are required to establish how levels of intermediate glycolytic metabolites change in CCA so that metabolic profiles of CCA and HCC can be adequately compared.

### Glycolysis: summary and future directions

Metabolic alterations towards aerobic glycolysis in PLC are reflected in the increased expression of glucose and lactate transporters (GLUT and MCT families, respectively), as well as in the upregulation of glycolytic enzymes. Overexpression of high-affinity glucose hexokinase HK2 and glycolysis-promoting ALDOA is a characteristic feature of both HCC and CCA. Moreover, prevalent in healthy liver tissue, isoform ALDOB, which acts as a tumor suppressor, is significantly downregulated in HCC. The expression of PKM2, a pyruvate kinase isoform with reduced enzymatic activity localized in both the cytoplasm and nucleus of cells, is elevated in PLC cells. The predominance of the PKM2 isoform leads to the accumulation of intermediate glycolysis metabolites necessary for cell growth and proliferation, while nuclear localization of the enzyme serves as a transcription regulator. Indeed, increased level of G6P in HCC and G6PD overexpression in both HCC and CCA suggest an elevated contribution of PPP in glucose metabolism. The increased lactate production favors G6P accumulation and activation of PPP and other pathways required for biomass synthesis and oxidative stress combating. Nevertheless, excessive lactate is especially vital for tumor growth as it helps fuel tumor cells via pyruvate regeneration for further OXPHOS in a hypovascular environment.

Despite a notable amount of information on glucose metabolism in PLC accumulated so far (Figure 1), several significant gaps need to be filled to improve our understanding of metabolic alterations in НСС and CCA cells. F6P to F1.6BiP transformation by PFK should be investigated not only as part of glycolysis but jointly with PFKBF3 and F2.6BiP, as F6P is an essential player in PPP. Thus, alterations in F6P concentrations upon the occurrence of CCA and HCC, as well as the regulation of enzymes associated with F6P, is a crucial task for understanding the role of this segment of glycolysis in the development, reoccurrence, and metastases of CCA and HCC. Besides, it is noteworthy to assess altered levels of 3PG and 2-phosphoglycerate (2PG). 3PG is an intermediate metabolite in the glycolytic pathway but can also directly participate in the serine synthesis pathway. In HCC, a decrease in the levels of these metabolites has been observed compared to healthy liver tissues. However, similar studies have not been conducted for CCA. Additionally, the expression of GAPDH and PGK1 is elevated in HCC, whereas in large-duct type CCA, a decrease in their expression has been noted. A more in-depth examination of these discrepancies, as well as verification of the results in other types of CCA would help understand PLC carcinogenesis better, especially in the case of cHCC-CCA.

## Citric acid cycle is a cross-road of energy metabolism and lipids accumulation

**FIGURE 2:**
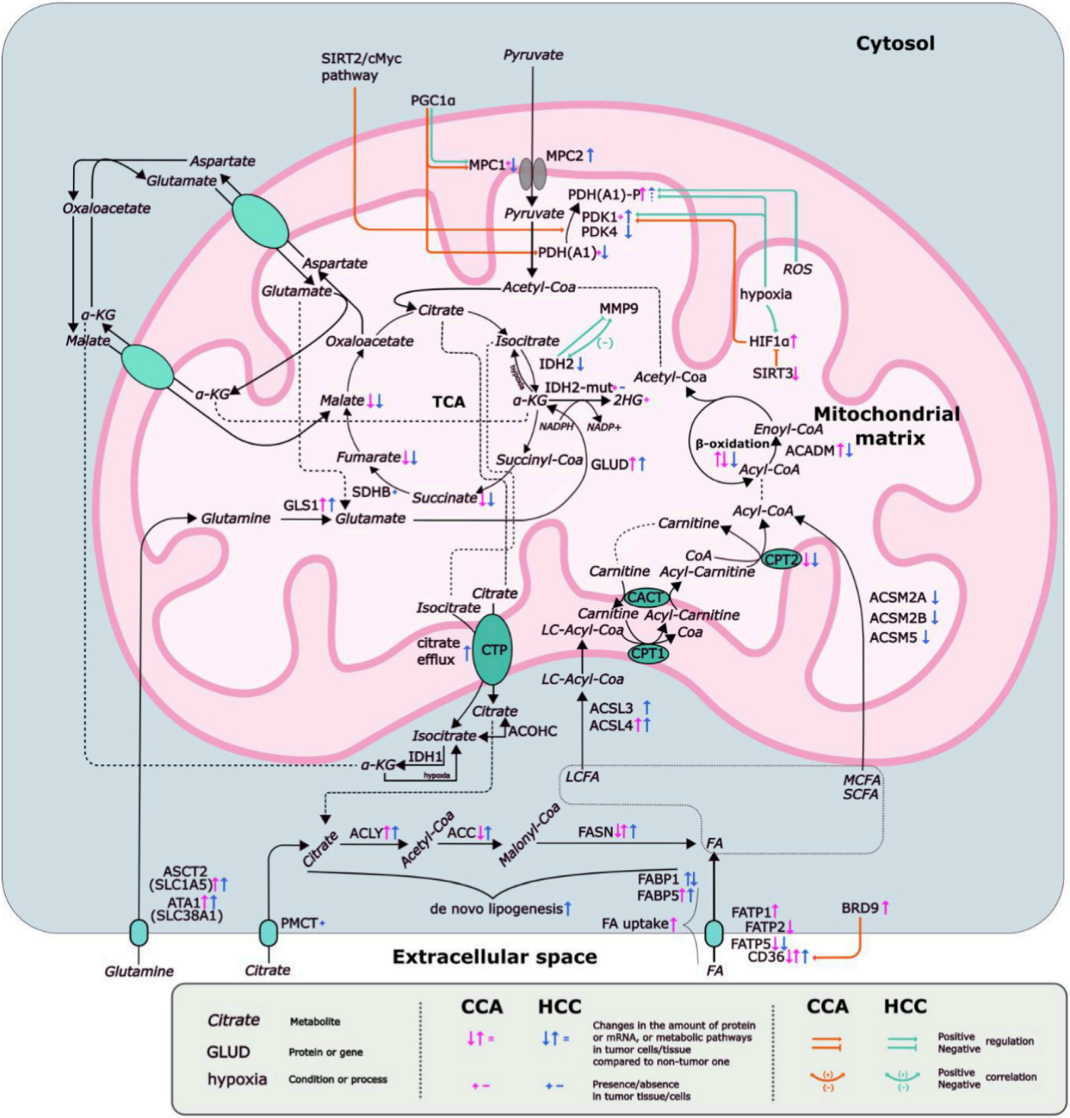
Alterations in mitochondrial and cytosolic energy metabolism in cholangiocarcinoma and hepatocellular carcinoma: TCA cycle, *de novo* lipogenesis and β-oxidation.

In his studies, Otto Warburg hypothesized that cancer cells prefer to perform aerobic glycolysis instead of cellular respiration due to possible dysfunctions in the citric cycle and the respiratory chain in tumor cells. This hypothesis was later disproved (Koppenol et al., 2011), which, in fact, does not mean that specific alterations in TCA-related pathways are absent in PLC cells. The general changes in mitochondrial and cytosolic energy metabolism are summarized in Figure 2.

### Pyruvate as a mitochondrial fuel

#### Pyruvate uptake

Regardless of the overall metabolic shift towards aerobic glycolysis, OXPHOS remains a vital energy source in PLC cells. Thus, pyruvate, either provided by glycolysis or produced from absorbed lactate by LDH, should be transported into mitochondria. So, the pyruvate transporter comes into play—the mitochondrial pyruvate carrier (MPC), which consists of two subunits, MPC1 and MPC2 (McCommis and Finck, 2015). In iCCA, decreased MPC1 expression correlates with poor survival prognosis, although the association was not statistically significant in the case of OS (Ohashi et al., 2018). At the same time, MPC1 downregulation correlates with levels of CA19-9, vascular invasion, and distant metastases, which may emphasize the close association of MPC1 expression with EMT and tumor progression. MPC1 expression decreases under EMT conditions, and the knockdown of MPC1 itself leads to EMT induction and a decrease in ROS levels. Moreover, overexpression of MPC1 suppresses the migration ability of the tumor (Ohashi et al., 2018). The regulator of MPC1 in CCA can be the peroxisome proliferator-activated receptor γ coactivator-1α (PGC1α), which, in combination with NRF1, binds to the MPC1 promoter, facilitating gene transcription and increasing MPC1 expression at both the mRNA and protein levels. Thus, increased flow of pyruvate into mitochondria promotes OXPHOS and consequently facilitates the invasion and migration of CCA tumor cells (Li et al., 2018).

A similar regulatory mechanism was observed in HCC, where the PGC1α/NRF1 complex binds to the MPC1 promoter, thereby regulating MPC1 expression (Wang C. et al., 2021). In both CCA and HCC, the PGC1α/NRF1-MPC1 axis, upon PGC1α overexpression, activates mitochondrial biogenesis and promotes the production of reactive oxygen species (ROS). However, unlike in CCA, where this fact increases migration and invasion without impacting proliferation level, in HCC, the increase in ROS leads to reduced proliferation and the induction of apoptosis. This may indicate stronger antioxidant mechanisms in CCA to maintain intracellular ROS homeostasis (Li et al., 2018; Wang C. et al., 2021). Wang C. et al. (2021) note that low levels of MPC1 and PGC1α are associated with poor prognosis in HCC patients, which aligns with the identified role of the PGC1α/NRF1-MPC1 axis in hepatocarcinogenesis. It is also important to mention that in the investigation of metabolic gene changes in HCC, upregulation of MPC2 and downregulation of MPC1 were observed in tumor tissues from HCC patients, indicating distinct roles for MPC1 and MPC2 in cancer development, but further research is needed to establish these roles (Nwosu et al., 2017).

#### Pyruvate decarboxylation

Once pyruvate enters the mitochondria, it undergoes decarboxylation to acetyl-coenzyme A (acetyl-CoA) by pyruvate dehydrogenase complex (PDC) to be utilized in TCA. PDC contains three main catalytic components: pyruvate dehydrogenase E1, dihydrolipoamide acetyltransferase E2, and dihydrolipoamide dehydrogenase E3. E1 and E2 are responsible for acetyl-CoA, while E3 reduces NAD^+^ to NADH (Patel et al., 2014). Regulation of PDC activity occurs through phosphorylation and dephosphorylation of the E1a subunit (PDHE1a/PDHA1), where phosphorylation of E1a at three serine sites leads to inactivation of the entire enzyme. Pyruvate dehydrogenase kinases (PDKs) are responsible for the phosphorylation of PDH, and various PDK isoforms have different catalytic activity to the E1a subunit (Saunier et al., 2016). The phosphorylation level of PDHA1 is elevated in both CCA cell lines and tumor tissues of CCA patients compared to adjacent tissues. Moreover, a high level of PDHA1 phosphorylation is associated with lower patient survival (Xu et al., 2019a). Being a SIRT2/cMYC pathway target, PDHA1 phosphorylation reduces oxygen consumption and significantly contributes to the energy metabolism reprogramming of CCA cancer cells. Moreover, the authors found another, no less important function of the SIRT2/cMYC pathway in CCA. According to the same study, the SIRT2/cMYC pathway contributes to enhancing the serine synthesis pathway (SSP) and antioxidant production, which ultimately allows CCA cells to reduce the level of oxidative stress-induced apoptosis (Xu et al., 2019a). The same authors proposed another mechanism of PDHA1 phosphorylation regulation in CCA: Sirtuin-3 (SIRT3) can inhibit the HIF-1α/PDK1/PDHA1 pathway, thereby contributing to the anti-Warburg effect. The study showed that HIF-1α expression and PDHA1 phosphorylation levels are elevated, while SIRT3 is downregulated compared to adjacent tissue. SIRT3 upregulation in CCA cell lines under hypoxic conditions leads to reduced expression of HIF-1α, PDK1, and decreased PDHA1 phosphorylation level, while SIRT3 downregulation has the opposite effect. Conversely, HIF-1α overexpression increases PDK1 expression and PDHA1 phosphorylation level in CCA cells. Thus, by reducing PDK1 expression in an HIF-1α-dependent manner, SIRT3 can maintain PDH in an active form, promoting ROS production, enhancing apoptosis, and reducing CCA cell proliferation (Xu et al., 2019b). Also, PGC1α in complex with NRF1 binds to the PDHA1 promoter, thereby increasing PDHA1 mRNA and protein levels. This contributes to enhanced mitochondrial metabolism, CCA invasion, and migration (Li et al., 2018).

In HCC, PDHA1 is downregulated in tumor tissue compared to adjacent normal tissue, both at protein and mRNA levels. Moreover, the reduced PDHA1 level is associated with lower OS of patients (Sun et al., 2019). Artificial upregulation of PDHA1 gene expression in HCC cells leads to a significant increase in PDH activity with a consequent increase in ATP production, but at the same time, reduces the production of lactate and glucose uptake. Additionally, PDHA1 overexpression causes a decrease in the proliferative capacity of HCC cells and enhances apoptosis through regulation of the intrinsic apoptosis pathway (Sun et al., 2019). These findings may indicate a significant role of PDHA1 in regulating glycolytic flux and, consequently, the metabolic phenotype of HCC cells. As previously noted, PDH is regulated by the PDK family. In the study by Xu et al., PDK1 expression was found to be elevated in HCC compared to normal liver tissue, and high kinase expression was associated with lower OS in HCC patients. Moreover, PDK1 inhibition by dicoumarol leads to a significantly reduced PDH phosphorylation and decreased glycolysis, which is reflected in reduced relative glucose uptake and lactate production, and enhanced OXPHOS. Interestingly, this does not lead to tumor growth and metastasis, which can be expected with a slightly increased level of ROS production, but also promotes apoptosis (Xu et al., 2020). Unlike PDK1, which is elevated in HCC, PDK4 expression, on the contrary, is significantly reduced in HCC tumor tissues compared to non-tumor liver tissues at both protein and mRNA levels. PDK4 downregulation is associated with a lower patient survival rate and a shorter time to recurrence. PDK4 silencing leads to increased proliferation and easier migration and invasion of tumor cells *in vitro*, while knockdown leads to induction of lipogenesis by enhancing the expression of fatty acid synthase (FASN) and SCD, key lipogenesis enzymes (Yang et al., 2019). A similar result was obtained in later work, where endogenous depletion of PDK4 in HCC promotes tumor development *in vivo* and proliferation *in vitro*, while PDK4 knockdown leads to increased migration and invasion of HCC cells, which proves the pro-tumor role of PDK4 downregulation in HCC (Qin et al., 2020). Interestingly, the effect of PDK4 on mitochondrial function is still ambiguous. In the study of Yang et al., PDK4 knockdown does not yield glycolysis or OXPHOS rates change in HCC cells (Yang et al., 2019), while more recent work clearly shows an increase in mitochondrial activity and energy metabolism in HCC cells after PDK4 knockdown (Si et al., 2021). Thus, the role of PDK4 in the development of HCC remains to be clarified. Extensive phosphorylation of PDH at all three serine sites is observed in HCC cells even under the early stage of hypoxia. Moreover, as early as 2 h after the start of exposure to hypoxic conditions on HCC cells, the first signs of PDH phosphorylation are observed simultaneously with the first expression of HIF-1α and HIF-2α proteins, while PDK1 mRNA is activated only after 4–6 h, and PDK1 protein induction occurs even later. Despite this, as shown in Zimmer’s work, PDHA1 phosphorylation does not depend on either HIF-1α or HIF-2α in hypoxia, but the knockdown of all four PDK isoforms leads to a sharp decrease in PDH phosphorylation levels. Thus, PDKs, but not HIF-1/2α, mediate the early phosphorylation of PDHA1 under hypoxic conditions (Haan et al., 2016). Remarkably, hypoxic conditions also cause an increase in the redox state of mitochondria after 2–3 h at the same time as pPDHA1 is observed, and artificially induced ROS under normoxic conditions in HCC cells increase PDH phosphorylation, which may indicate the potential role of ROS in stimulating PDC phosphorylation (Haan et al., 2016).

### Citric acid cycle

MPC1 downregulation and PDH inhibition not only reduce mitochondrial respiration activity but could also affect the levels of TCA metabolites utilized for lipid synthesis and other vital cellular pathways. There was a significant reduction in fumarate, malate, and succinate levels in both HCC (Huang et al., 2013) and CCA (Kitagawa et al., 2023) tissues. The functioning of the Krebs cycle and, consequently, the production of the corresponding metabolites can be influenced not only by the level of pyruvate that enters the mitochondria, but also by several changes that occur in the TCA cycle itself. In 10%–20% of CCA cases and 5%–10% cHCC-CCA cases (Boscoe et al., 2019; Hong et al., 2026), mutations are observed in IDH genes (IDH1, IDH2), which encode isocitrate dehydrogenase 1 and 2 - cytosolic and mitochondrial enzymes responsible for the oxidative decarboxylation of isocitrate to α-ketoglutarate (αKG) (Boscoe et al., 2019; Reitman and Yan, 2010; Kipp et al., 2012; Dorovatovskaia et al., 2025). Interestingly, the mutation occurs more frequently in intrahepatic cholangiocarcinoma than in extrahepatic cholangiocarcinoma and is not directly related to the overall survival rate of patients with CCA (Boscoe et al., 2019; Kipp et al., 2012). The mutant enzyme catalyzes an irreversible conversion of αKG into 2-hydroxyglutarate (2HG) and consumes NADPH generated during αKG formation from isocitrate or glutamate (Han et al., 2020; Pirozzi and Yan, 2021). The presence of 2HG and decreased αKG level have a strong pro-tumor effect on cells (Dorovatovskaia et al., 2025), affecting energy reprogramming, accumulation of reactive oxygen species, and dependence on glutamine. In the study of Delahousse et al., 2HG was investigated as a marker of iCCA and it turned out that the level of the D-2HG enantiomer correlates with tumor burden and response to IDH-targeted therapies or indirect therapies (Delahousse et al., 2018).

IDH mutations were not detected in HCC, but several single nucleotide polymorphisms in transcription factors binding sites (both for IDH1 and IDH2) or one splicing site in the IDH2 gene can be significantly associated with the risk of death of HCC patients (Zhang et al., 2014). The expression level of the IDH2 enzyme is reduced in HCC tumor tissue compared to paracarcinoma tissue and decreases even more with the formation of distal metastases. HCC patient with low level of IDH2 have a poorer five-year survival rate than patients with high levels of the enzyme, and the level of IDH2 expression in HCC tissues has a significant inverse correlation with matrix metalloproteinase 9 (MMP9) level, a known control factor for cellular metastasis. By inhibiting MMP9, IDH2 suppresses cell invasion. Thus, the downregulation of IDH2 leads to tumor invasion, metastasis, and development (Tian et al., 2015). Succinate dehydrogenase B (SDHB), an enzyme that catalyzes the oxidation of succinate to fumarate, is also often lowered in HCC cells and tumor tissues (Tseng et al., 2018). HCC patients with low level of SDHB expression have poorer DFS and OS. Knockdown of SDHB in HCC cells leads to a switch in the tumor phenotype from aerobic respiration to glycolysis and a significant increase in cell proliferation and migration, which is consistent with the results of an *in vivo* study, where SDHB knockdown led to an increase in tumor volume and accelerated metastasis (Tseng et al., 2018).

#### Short summary on TCA

TCA enzymes make an important contribution to developing optimal conditions for PLC development and progression. Unfortunately, there is a lack of knowledge to fully characterize mitochondrial function, particularly the TCA cycle in HCC and CCA, and to observe differences between these diseases. Nevertheless, it can be concluded that mitochondrial function in both diseases changes significantly, which is accompanied by alterations in cancer cell energy metabolism, redistribution of mitochondria’s and glycolytic pathway contributions to energy metabolism, and consequently, the formation of an optimal metabolic network within cells to support tumor viability and development.

### Glutamine fueling

Rapidly proliferating cells have high demands for building blocks and energy for development. Apart from glucose, cancer cells rely on glutamine as a vital source of matter and energy for survival (Jin et al., 2023; Hosios et al., 2016). Glutamine is a nonessential amino acid synthesized in cells *de novo* by glutamine synthase (GS) (Yang et al., 2016). In proliferating cells, glutamine plays one of the key roles as a precursor to the synthesis of glutamate, proline, aspartate, and asparagine. In addition, glutamate serves as a nitrogen source for the synthesis of alanine, serine and glycine. Glutamine also promotes cell proliferation by being a nitrogen donor for the synthesis of purine and pyrimidine nucleotide bases (Zhang et al., 2017). Glutamine is transported into the cell through glutamine transporters ASCT2 (SLC1A5) or ATA1 (SLC38A1) and can then be converted to glutamate by glutaminase (GLS1/2). Glutamate, in turn, serves as a source of nitrogen and carbon for the synthesis of amino acids and nucleic acids. In addition, glutamate can be converted to αKG by mitochondrial glutamate dehydrogenases (GLUD), thereby becoming a direct participant in TCA (Park et al., 2020). Moreover, reductive carboxylation of αKG becomes an essential pathway for citrate synthesis under hypoxic conditions (Mullen et al., 2014; Wise et al., 2011), which supports acetyl-CoA generation from cytosolic citrate for further *de novo* lipogenesis.

In hCCA, significant overexpression of ATA1 is observed in tumor tissue compared to normal and para-neoplastic bile ducts. The ATA1 expression level correlates with lymph node metastases and disease stage. Moreover, ATA1-positive patients are associated with increased recurrence rate after tumor resection (Yu et al., 2011). Additionally, increased expression of the ASCT2 is observed in CCA tumor tissue compared to paracarcinoma tissue (Ni et al., 2023), which collectively indicate increased glutamine uptake by CCA cells.

In iCCA, the GLS1 protein level is also elevated in tumor tissue compared to adjacent normal tissue. GLS1 expression levels determine the cells’ ability to migrate, invade, and undergo the epithelial-mesenchymal transition process *in vitro*. GLS1 protein overexpression is associated with lymphatic metastases, poor tumor differentiation, and poor OS of patients, making GLS1 an independent predictor of OS and cumulative recurrence in iCCA (Cao et al., 2019). GLUD expression is also increased in extrahepatic CCA tissues compared to normal bile duct tissues and correlates with the degree of cell differentiation, depth of vascular invasion, lymph node metastasis and neural invasion. The median survival rate of extrahepatic CCA patients with low level of GLUD is significantly higher (26 months versus 11.6 months), compared to patients with upregulated enzyme. At the same time, tumor cell proliferation, migration, and invasion become significantly reduced, and the apoptosis rate increases upon GLUD silencing (Su et al., 2017).

According to a study of The Cancer Genome Atlas (TCGA) and a Clinical Proteomic Tumor Analysis Consortium (CPTAC) databases, in HCC, there is a significant increase in mRNA levels of both ATA1 and ASCT2 (the latter was also confirmed at protein level) in tumor tissue compared to paracarcinoma tissue, which also correlates with the clinical stage, tumor size and is associated with poor OS, DFS and other clinical and pathological characteristics of patients (Sun et al., 2016; Liu et al., 2021; Zhao et al., 2021).

Among the enzymes of the glutamine metabolic pathway, GLS1 glutaminase upregulation in HCC tumor tissue positively correlates with the presence of lymphatic metastases, late-stage TNM, and poor OS. GLS1 promotes cell proliferation, probably through the AKT/GSK3b/CyclinD1 pathway (Xi et al., 2019). In HCC tissues, there is also a two-fold increase in the level of GLUD1 mRNA expression compared with normal hepatocytes. GLUD1 silencing in HCC cell lines leads to a significant decrease in cell proliferation, while the opposite effect is observed in healthy hepatocytes. In addition, GLUD1 silencing in HCC cells leads to activation of internal apoptosis and degradation of mitochondria, which makes GLUD1 a potential target for HCC therapy (Marsi et al., 2021).

#### Summary: the importance of glutamine metabolism in PLC

Both PLCs adopt glutamine fueling by significantly upregulating glutamine transporters and subsequent metabolic pathway enzymes, which indicates glutamine is an essential resource for TCA cycle functioning and further lipogenesis.

### Lipid fueling

The ability to switch energy metabolism from OXPHOS to fatty acid oxidation (FAO) is an established hallmark of cancer cells (Ma et al., 2025). The aerobic breaking down of endogenous fatty acid (FA) complements anaerobic lactate formation from pyruvate, enhancing energy production in mitochondria. Moreover, in the cases of glucose deficiency caused by tumor hypovascularization, cancer cells activate FAO to utilize triacylglycerols, previously reserved in lipid droplets inside cells, to maintain energy homeostasis (Wu et al., 2020). Prior to β-oxidation, FA must pass through two mitochondrial membranes. Long-chain fatty acids (LCFAs) and medium-chain fatty acids (MCFAs) longer than eight carbon atoms utilize the carnitine shuttle. In contrast, short MCFAs and short-chain fatty acids (SCFAs) freely penetrate mitochondrial membranes by carnitine-independent uptake. SCFAs and MCFAs are further activated by ACSL to the corresponding acyl-CoA thioesters to enter FAO and subsequent TCA cycle (Schönfeld and Wojtczak, 2016).

In HCC, a significant increase in ACSL3 and ACSL4 protein expression is observed in liver tumor tissue compared to healthy or CCA tissues. ACSL4 protein expression is higher in CCA than in the control group, while the ACSL3 level was not altered between control and CCA samples.The localization of both ACSL3 and ACSL4 is associated with lipid droplets and the endoplasmic reticulum, but ACSL4 is also partially located on the plasma membrane of HCC cells (Liu et al., 2023; Gao et al., 2017; Ndiaye et al., 2020). Also, a correlation was shown between high ACSL4 expression level in CCA patients and poor OS. So, ACSL4 knockdown leads to a decrease in CCA cell proliferation and migration (Liu et al., 2023), probably due to the vital role of ACSL4 in fatty acids *de novo* synthesis and further lipogenesis. Along with the increased expression of long-chain acyl-CoA synthetases (ACS), a significant decrease in the expression of mitochondrial medium-chain acyl-CoA synthetases ACSM2A, ACSM2B, and ACSM5 is observed in HCC tissues and cells compared to non-tumor samples, indicating abnormal fatty acid metabolism in HCC. Moreover, low expression level of ACSM5 is associated with poor overall survival of patients, while overexpression of this enzyme in the Huh7 cell line reduces fatty acid accumulation and suppresses tumor growth (Yang et al., 2024). The underexpression of mitochondrial ACS indicates a reduced role of OXHOS and, broadly, mitochondrial function in the energy metabolism of HCC cells through deregulation of the mitochondrial fatty acid synthesis pathway.

Significant downregulation of carnitine palmitoyltransferase-2 (CPT2) is observed both in HCC and CCA tumors compared to adjacent non-tumor tissues (Fujiwara et al., 2018; Jiang et al., 2023). The CPT2 expression level correlates with CCA patients survival, which is explained by the ability of CPT2 overexpression to inhibit the TNFα/NF-κB signaling pathway, thereby suppressing CCA progression (Mao et al., 2025). ASCL4 and carnitine palmitoyltransferase-1A were upregulated in tumor tissues obtained from a diethylnitrosamine-induced mouse model of HCC on a high-fat diet, while CPT2 was downregulated. The accumulation of acylcarnitines in tumor tissue obtained from animals on a high-fat diet indicates reduced beta-oxidation, which could be an adaptive mechanism for HCC in a lipid-rich environment to avoid lipotoxicity. Indeed, CPT2 knockdown in mouse HCC cells leads to cancer cell resistance to lipotoxicity (Fujiwara et al., 2018).

Medium-chain acyl-CoA dehydrogenase (ACADM), one of the key enzymes in FAO, is downregulated in human HCC tumor tissue. Its expression is inversely correlated with larger tumor size, presence of invasion, and late disease stage. ACADM knockdown leads to reduced FAO, accumulation of intracellular lipids, triglycerides and phospholipids, enhanced cell proliferation and growth, metastasis, and invasiveness (Ma et al., 2021). Conversely, an increase in ACADM expression was observed in CCA cell lines compared to normal human cholangiocytes. Similarly, significant ACADM expression was observed in both the stroma and epithelium of iCCA tumors, although its expression in normal bile duct tissue was negligible (Ruiz de Gauna et al., 2022). The correlation between the expression of ACADM and the proliferating cell nuclear antigen marker in iCCA tissues indicates a link between FAO and the level of tumor proliferation: An increase in FAO rate was observed only in highly-proliferative EGI1 cells, while the FAO rate in the low-proliferative HUCCT1 cell line was significantly reduced compared to normal bile duct cell lines (Ruiz de Gauna et al., 2022).

#### Short summary on β-oxidation

Highly proliferative CCA cell lines, in contrast to HCC cells, implement FAO to satisfy high energy requirements for proliferation and invasion. Nevertheless, additional studies of the effect of FAO on HCC cell proliferation and disease development are required separately in the case of low- and high-proliferative cells to uncover whether FAO decrease is a general trend in HCC or a feature of the specific cell line.

### Citric acid cycle: summary

Despite the overall reduced mitochondrial activity, TCA remains vital for balancing energy and biomass pathways in PLC cells. The decreased availability of pyruvate and PDH inhibition through its intensive phosphorylation reduces acetyl-CoA production and, thus, TCA fueling. Thus, increased glutamine uptake and its further conversion to aKG increase their contribution to energy production in mitochondria and show the demand to avoid significant mitochondrial impairment. FAO, an alternative source of acetyl-CoA for the TCA cycle, does not play a substantial role in energy production in PLC cells, with the exception of highly-proliferative CCA cells with enormous energy demands. However, the detailed role of FAO in PLCs in nutrient-deficient conditions has still not been investigated.

## Lipid portrait of primary liver cancers

Lipid composition changes dramatically in proliferative cells, which reflects on the balance of various lipid classes and, especially, in their fatty acid composition. Fatty acids are essential metabolites of the cell that are stored as TAGs in intracellular lipid droplets, incorporated in polar lipids as building blocks for membranes, and also play a role in signaling. In addition, FA can be oxidized to produce acetyl-CoA to support TCA or synthesized if an excess of TCA metabolites emerges.

### TCA metabolites as a source of carbon skeleton for lipid synthesis

Citric cycle enzymes and their isoenzymes are an important source of intermediates for biomass accumulation, especially lipid synthesis, in proliferative cells. Mitochondrial citrate can be transported to the cytosol by citrate transport protein (CTP, SLC25A1) and converted back to acetyl-CoA for further *de novo* lipogenesis (Sun et al., 2010). Isocitrate, which is also excreted to the cytosol by CTP, can be converted either to citrate by aconitase 1, or to glutamine through subsequent action of cytosolic IDH, GLUD, and GS (Mosaoa et al., 2021). In healthy liver tissue, a high expression of CTP mRNA is necessary to support gluconeogenesis and lipid synthesis (Huizing et al., 1998). At the same time, according to the Human Protein Atlas, high expression of CTP is also observed in liver cancer (Poolsri et al., 2018). Kaplan et al. (1982) found that the mitochondria of the rapidly growing poorly differentiated HCC 3924A cell line have a significantly higher flow of citrate from the mitochondria into the cytoplasm, which is associated with a “compensatory effect” of reducing the number of mitochondria to 60% in this cells. Even in the case of a slow-growing, well-differentiated HCC 16 cell line, an increased rate of citrate flow rate between mitochondria and cytosol compared with healthy hepatocytes is observed (Kaplan et al., 1982). Mitochondrial CTP inhibition leads to a significant decrease in HCC cell viability and induction of apoptosis with a simultaneous increase in ROS formation and suppression of Bcl-2 activity in tumor cells, which may also explain increased apoptosis. Interestingly, inhibition of plasma membrane citrate transporter (PMCT) protein, an important player in the transfer of citrate from the extracellular environment as an alternative way to increase citrate level in the cytoplasm, leads to the same effects, and the combination of these inhibitors further contributes to the cytotoxic effect against cancer cells even at lower citrate concentrations. Treatment of primary hepatocytes with CTP and PMCT inhibitors, as well as their combination, does not lead to liver cell apoptosis, which makes these inhibitors a convenient means for treating liver cancer (Poolsri et al., 2018). As expected, citrate transporters inhibition in HCC cells leads to a noticeable suppression of the *de novo* lipid synthesis and, as a consequence, a reduction in intracellular free fatty acid content without affecting the expression of the main lipogenesis enzymes such as FASN, acetyl-CoA carboxylase (ACC), ATP citrate lyase (ACLY) (Poolsri et al., 2018). A significant role of CTP in maintaining the viability of HCC cells is confirmed by the survival analysis of HCC patients from the TCGA database, where it was found that patients with high SLC25A1 expression (CTP) have significantly lower OS (You et al., 2023). To the best of our knowledge, no studies have been conducted that could characterize the work of the citrate transporter in CCA, or reveal corresponding changes relative to healthy tissue.

Besides citrate, αKG can serve as a carbon skeleton source for *de novo* lipid synthesis (Mullen et al., 2014; Wise et al., 2011) after exporting from the mitochondria to the cytoplasm through the malate-α-ketoglutarate transporter of the malate-aspartate shuttle (Scalise et al., 2016; Lu et al., 2008). αKG can be formed both as a result of glucose metabolism or from glutamine (Scalise et al., 2016), which gives cells the opportunity to adjust their metabolic pathways and maintain lipid synthesis. To the best of our knowledge, there have been no studies dedicated to αKG and its transfer from mitochondria to the cytoplasm in HCC and CCA. However it can be assumed that with possible disruption of citrate transport, αKG will serve as an alternative carbon skeleton source for the fatty acid synthesis necessary for proliferative cells.

### Where do the fatty acids come from?

The main enzymes of the *de novo* fatty acids synthesis pathway are ACLY, ACC, and FASN, which sequentially convert citrate to acetyl-CoA, malonyl-Coa, and palmitate, respectively (Ameer et al., 2014). In human HCC, an increased rate of *de novo* lipogenesis is observed, which is reflected in the high expression of mRNA and proteins ACLY (Han et al., 2021; Calvisi et al., 2011; Yahagi et al., 2005), ACC (Calvisi et al., 2011; Yahagi et al., 2005), and FASN (Calvisi et al., 2011; Yahagi et al., 2005) in tumor tissue compared with non-tumor liver tissue. Enhanced FAs synthesis in HCC, which is supported by increased CTP expression, is essential for tumor survival, as cell viability reduction occurs upon CTP inhibition (Poolsri et al., 2018). Thus, HCC cells are significantly dependent on *de novo* lipogenesis, which in turn relies on the availability of citrate—one of the products of glucose metabolism. The relationship between glucose consumption level and lipid content has also been noted in the study by Wang et al., where a positive correlation between these two parameters was observed in both HCC cells and HCC patients tissues (Wang et al., 2016). Thus, glucose metabolism and lipogenesis are two closely linked metabolic pathways essential for the viability and development of HCC.

*De novo* synthesis is not the only source of FAs for further lipogenesis. However, fatty acid transport protein-5 (FATP5), a tissue-specific transporter for LCFA in normal hepatocytes (Anderson and Stahl, 2013), is notably downregulated at mRNA and protein levels in HCC tumor (Wang M. D. et al., 2021). Furthermore, low expression of FATP5 is associated with poor survival outcomes for HCC patients and tumor aggressiveness. Interestingly, the proposed mechanism by which FATP5 influences EMT and HCC metastasis includes a reduction in glucose metabolic flux at high levels of FATP5, which in turn leads to decreased metastasis and EMT via the AMPK-mTOR-S6K-S6 pathway (Wang M. D. et al., 2021). Thus, the loss of FATP5, despite the important role of this transporter in FAs uptake, is beneficial for HCC cells. In contrast, the expression of the fatty acid receptor CD36, which also actively participates in the LCFA uptake, facilitating LCFA transport into cells (Pepino et al., 2014), is significantly elevated in HCC tumor tissue compared to non-tumor liver tissue. CD36 knockdown leads to suppressed proliferation, migration, and invasion of HCC cells *in vitro*, while overexpression produces the opposite effect, promoting tumor growth *in vivo*. Furthermore, significant upregulation of CD36 mRNA expression is observed in HCC cell lines compared to normal hepatocytes, although this trend at the protein level is not consistent across all hepatoma cell lines (Luo et al., 2021). But what is more important within the context of this review is that CD36 overexpression induces an increase in glycolytic flux and lactate production in HCC cells through the Src/PI3K/AKT/mTOR signaling pathway while having little effect on oxygen consumption levels. This suggests a significant role of CD36 in HCC not only as a facilitator of FAs uptake by tumor cells but also as a stimulator of aerobic glycolysis (Luo et al., 2021).

Fatty acid-binding proteins (FABPs) also participate in the regulation of FAs uptake and transport (Owada, 2008; Hotamisligil and Bernlohr, 2015). FABPs are tissue-specific proteins widely expressed in tissues with intensive FA metabolism. Among the ten types of these proteins found in humans, FABP1, FABP2, and FABP5 are observed in healthy liver tissue, although this profile can change significantly in the presence of pathology (Smathers and Petersen, 2011). A significant downregulation of FABP1 is typical for HCC tumor tissue compared to paracarcinoma ones, and the amount of stored TAGs in the HCC tissue is also reduced. Induced FABP1 overexpression in HCC cells inhibits the migration and proliferation of tumor cells *in vitro*. It also reduces the level of ROS, which, by itself, negatively affects the level of metastasis and invasion (Lin et al., 2021). Thus, FABP1 plays a tumor-suppressive role, and considering its critical importance in LCFA uptake by cells and their intracellular transport (Wang G. et al., 2015), its decrease in HCC tissues protects cells from excessive lipid accumulation to avoid lipotoxicity amid already high levels of *de novo* FA synthesis. Additionally, a decrease in FABP1 expression helps maintain sufficient ROS levels necessary for tumor progression. Interestingly, an earlier study reported the opposite observation: an increase in FABP1 level in HCC tissues compared to adjacent liver tissues (Ku et al., 2016). The discrepancy originated from differences in patient characteristics between studies, such as concomitant diseases, the origin of HCC (a history of fatty liver disease), and, possibly, patients’ body mass index. So, further studies of FABP1 in HCC pathogenesis are required to establish its precise role in this process. In HCC, a significant upregulation of FABP5 in tumor tissue is also observed compared to non-tumor control, and a high level of the expression correlates with a lower OS and DFS (Li et al., 2024b). Interestingly, no difference in FABP1 expression was found between tumor and non-tumor tissues in this study, indicating a complex and ambiguous role of FABPs in HCC, and this issue requires further investigation. Thus, taking into account changes in the pathway of *de novo* FAs synthesis in HCC, as well as alterations in the transport of exogenous FAs into cancer cells, it can be concluded that in HCC, both methods of obtaining fatty acids by cells take place, but *de novo* synthesis still prevails.

The opposite scenario is observed in the case of CCA, where tumor cells depend more on the consumption of extracellular fatty acids rather than intracellular synthesis. First, this is reflected in the expression of key lipogenesis enzymes. In CCA, a notable decrease of FASN and ACC at mRNA and protein levels is often observed in human CCA tumor tissue compared to healthy control (Li et al., 2016). Furthermore, it has been shown *in vivo* that ablating FASN in the mouse liver does not affect AKT/NICD-induced iCCA formation, whereas the development of HCC is heavily dependent on the presence of this enzyme (Li et al., 2016). However, it is not so unambiguous: The study by Tomacha et al. (2021) showed that high expression of FASN in CCA tissues significantly correlates with advanced disease stage and shorter OS. Furthermore, FASN knockdown leads to the inhibition of growth, migration, and invasion of CCA cells, as well as cell cycle arrest and stimulation of apoptosis. Thus, although FASN expression is not essential for CCA development, it plays an important role in tumor progression. The direct correlation between high FASN expression level and advanced-stage CCA may be explained by the developed nutrient deficiency and, thus, increased FA demand for further proliferation. Interestingly, unlike FASN and ACC, the expression of ACLY is significantly higher in CCA tumor tissue compared to paracarcinoma tissue. Patients with elevated ACLY level exhibit markedly poorer survival (Sun et al., 2024), confirming the increased contribution of fatty acid synthesis to the progression of CCA at advanced stages (Tomacha et al., 2021). ACLY overexpression also plays a crucial role in CCA resistance to ferroptosis, as inhibition of this enzyme promotes ferroptosis (Sun et al., 2024). Thus, a decrease in lipogenesis can be observed in CCA, but an increase in ACLY helps maintain at least a minimal level of lipogenesis, accelerating biomass synthesis and enhancing tumor aggressiveness.

In the context of reduced *de novo* FA synthesis, the FA requirements of CCA cells can be met by enhanced uptake of extracellular lipids. As observed by Gauna et al., the level of FA uptake is significantly higher in both low-proliferative CCA cell lines (HUCCT1) and high-proliferative ones (EGI1) compared to healthy cholangiocytes (NHC), but EGI1 cells exhibit a much higher level of fatty acid uptake. Interestingly, CD36 expression is elevated only in EGI1 cell line, while its downregulation was observed in other CCA cell lines with lower proliferation potential. However, the expression of FABP5, a regulator of fatty acid uptake and intracellular transport, was increased in all CCA cell lines (Ruiz de Gauna et al., 2022). One of the regulators of CD36 expression is bromodomain-containing protein 9 (BRD9), which is elevated in iCCA cells compared to non-tumor biliary cells. By binding to its promoter region, BRD9 directly activates CD36 transcription, thereby promoting enhanced cellular proliferation and progression of iCCA (Bu et al., 2025). Moreover, the expression of FABP5 is significantly higher in extrahepatic cholangiocarcinoma cells and hCCA cells compared to iCCA, which may suggest a role for FABP5 in the malignant progression specifically of large biliary tract cancer (Nakagawa et al., 2020). In mouse AKT/NICD-induced CCA model, the expression of SLC27A2 (which encodes FATP2) and SLC27A5 (FATP5) is markedly downregulated in tumor cells compared to non-tumor liver tissue. In contrast, the expression of SLC27A1 and lipoprotein lipase (LPL) are upregulated. This is consistent with results obtained from the TCGA dataset: the levels of SLC27A2 mRNA and SLC27A5 mRNA are downregulated, while SLC27A1 and LPL are elevated in human CCA samples compared to non-tumor liver tissue. Additionally, a downregulation in CD36 mRNA level in human CCA was noted (Li et al., 2016). A curious positive feedback loop of oleic acid-PPARγ-FABP4 was discovered by Zhang et al., where PPARγ, by stimulating fatty acid uptake through FABP4, promotes cholangiocarcinoma cell colonization in lymph node metastases microenvironment (Zhang H. et al., 2024). HCC cells, but not CCA, produce fatty acids through de novo synthesis. Thus, in contrast to HCC cells, CCA cells predominantly rely on the uptake of extracellular fatty acids, while *de novo* fatty acid synthesis is diminished; however, further studies are needed to understand better the regulatory processes governing extracellular fatty acid consumption.

### Impact on the lipid composition

Rearrangements of metabolic pathways, particularly, the pathways of absorption, synthesis, and metabolism of FA, affect the lipid composition of tumor cells and serve as both the cause and consequence of lipid metabolism alterations. The level of saturated fatty acids (SFA) in HCC tissues of PTEN-knockout mice is reduced compared to paracarcinoma tissues. PTEN, a cell cycle regulator and tumor suppression gene, is typically reduced or absent in at least half of human HCC cases, resulting in increased cell growth, survival, and migration rates. PTEN knockout yield in adopting pro-proliferative lipid portrait: various n3PUFAs and n6PUFAs with 18 carbon atoms chains become downregulated. In contrast, the levels of various n9MUFAs and n6PUFAs with 20 or 22 carbon atoms chains increased compared with the control. This trend can be observed simultaneously for phospholipids, cholesterol esters, and free fatty acids in both tissues and blood plasma of experimental animals (Muir et al., 2013), and is also consistent with the data obtained in the study of the xenograft model of HCC (Ma et al., 2023). The change in the lipid profile demonstrates an acceleration of fatty acid metabolism, which is confirmed at the level of the corresponding enzymes, namely, increased expression of FA desaturases Fads (Fads1, Fads2), Scd (Scd1, Scd2) and FA elongases Elovl (Elovl1, Elovl6) in HCC tissues compared with adjacent non-tumor tissues (Muir et al., 2013). Interestingly, in human HCC tissues an increase in free SFA (16:0, 18:0) was noted compared with non-cancerous liver tissue (Wang et al., 2023; Huang et al., 2013). This discrepancy is attributed both to differences in the nature of the tumor and to differences in the samples studied - the first of these studies was conducted on an animal model. At the same time, an increase in free MUFAs (16:1, 18:1, primarily n9) and a decrease in free PUFAs were noted in all studies. (18:2, 18:3, 20:3). Thus, the general trend on the substitution of ingested n3-unsaturated fatty acids and their PUFA-derivatives with *de novo* synthesized SFA and their n9- or n6-derivatives becomes clearly visible in the general case.

Generally, a decrease in ceramide levels and an increase in cholesterol levels is observed in HCC tissues as it helps tumor cells resist oxidative stress and apoptosis (Buech et al., 2020). Alterations in the FA composition of tumor cells affect the composition of membranes and triacylglycerols (TAG). In human HCC tissues, there is a decrease in the level of TAGs with the total number of double bonds >2 (except for 56:5 and 56:4 TAGs), while the concentration of other TAGs (except for 52:2), on the contrary, is significantly increased compared with para-carcinoma tissues (Li et al., 2017a). However, the unsaturation pattern of phospholipids is slightly different. The amount of phosphatidylcholines (PC), phosphoethanolamines (PE) and phosphatidylinositols (PI) with the number of double bonds equal to 0, 1, or 3 is increased, while a decrease in the amount of PC, PE, and PI with the number of double bonds equal to 2, 4 or 6 in HCC tissues compared to para-carcinoma tissues is observed. At the same time, the level of sphingomyelin (SM) is also increased in the tumor tissue, while the levels of ceramide (Cer) and phosphatidylglycerol (PG), on the contrary, are reduced (Li et al., 2017a). In another study comparing the lipids of human HCC tumor tissue with non-tumor tissue, a decrease in the levels of all types of ceramide was also confirmed, as well as an increase in the level of SM in tumor tissue. Moreover, there was also a decrease in PUFA-PC, PUFA-PE, phosphatidylserines (PS), especially PS 36:1 and PS 40:6, PI 38:4, PUFA-PI and an increase in Saturated-PC, Saturated-PE, cholesterol esters (CE), LysoPC 16:1, LysoPC 20:3 in HCC tumor tissue compared to adjacent non-tumor (Krautbauer et al., 2016; Kaplan et al., 1982). Several studies, based on untargeted metabolomics, including ambient ionization mass spectrometry, also demonstrate similar changes in the predominant FA and lipids in HCC tissues (Wang et al., 2023; Ma et al., 2023; Ferrarini et al., 2019). Although the overall trends are consistent across these studies, discrepancies in the levels of individual lipids may be attributed to limited sample sizes in some studies, as well as geographical differences in patient populations and the presence of cirrhotic changes in the liver. Thus, it can be noted that accelerated fatty acid metabolism, along with a simultaneous decrease of dietary fatty acid uptake, leads to a reduction in the proportion and degree of unsaturation of PUFAs in lipids of all classes in HCC tissues, which is especially noticeable for fatty acids with chain lengths of at least 20 carbon atoms, as well as n3 fatty acids, including n3-MUFAs.

Changes in the lipid profile have also been demonstrated in human CCA cells. A notable increase in the uptake of very low-density lipoproteins (VLDL) and high-density lipoproteins (HDL), as well as FA and CE, was observed in the highly proliferative CCA cell line compared to healthy cholangiocytes and low-proliferative CCA cells (Ruiz de Gauna et al., 2022). Additionally, elevated TAG levels and decreased PC levels were shown in both high- and low-proliferative CCA cells compared to healthy cholangiocytes despite the constant level of PC synthesis. Cer are also downregulated in human CCA cell lines, but the decrease is more pronounced in low proliferative cells. Moreover, an increase in SM levels was observed in low-proliferative, but not in highly proliferative cells, which is consistent with a decrease in sphingomyelinases activity in both cell lines and, conversely, an increase in SM synthase activity only in a low-proliferative cell line (Ruiz de Gauna et al., 2022). However, to the best of our knowledge, no studies have been conducted to date on changes in fatty acid composition in cholangiocarcinoma tissues. This information is necessary for a deeper understanding of the rearrangements of lipid metabolism, which can affect the degree of tumor malignancy and its proliferative potential.

### Lipid metabolism: summary and future directions

In contrast to glycolysis and the pathway of glutamine metabolism, where similarities in these processes are observed in both CCA and HCC, precise differences between nosologies occur in fatty acid metabolism. In HCC, upregulation of key enzymes involved in *de novo* fatty acid synthesis (CTP, ACC, FASN) indicates an elevated demand for newly synthesized FAs by HCC cells. Conversely, in CCA, FAs uptake is enhanced, suggesting that CCA cells rely more on extracellular FA pool rather than synthesizing FA within the cells. This divergence between HCC and CCA is attributed to differences in the environments of these tumors and FA availability. Moreover, HCC and CCA cells consume FA differently, which reflects specific energy and building block requirements. In particular, increased beta oxidation is observed in highly proliferative CCA cells, whereas FAO rate is inversely correlated with tumor severity in HCC. Thus, we determine characteristic distinctions in the ways of obtaining and using FA by tumor cells in PLCs, so studying the metabolism of fatty acids and lipids in cHCC-CCA becomes particularly interesting. The investigation of lipid metabolism in cHCC-CCA will help to understand this tumor origin and development, and, therefore, establish a more accurate classification to ensure the most effective treatment.

Although the metabolism alterations in HCC cells has been studied profoundly, it is worth paying attention to the “gaps” that make it difficult to understand the process of carcinogenesis fully. To the best of our knowledge, there have been no studies of changes in the citrate/isocitrate flux in CCA cells, including alterations in the expression of the corresponding transporters–PMCT and CTP. Such a study can provide an understanding of whether citrate plays a role in the development of CCA and how important it is for *de novo* lipogenesis in this disease. As noted earlier, αKG exported from mitochondria can serve as an alternative source of carbon skeleton for *de novo* lipid synthesis. Thus, the possible alterations in the malate-aspartate shuttle in both CCA and HCC as a part of a mechanism for αKG transfer between the mitochondria and the cytoplasm should be investigated.

## Conclusion

Both major PLCs share general alterations toward increasing energy production and biomass synthesis to support proliferation (Table 1). Increased lactate synthesis plays an essential role in glycolysis acceleration, but regulation of its bidirectional transporters appears to be even more significant. Despite the overall inhibition, TCA remains a vital source of building blocks for cell proliferation and provides energy through further OXPHOS. Due to the reduced transportation rate of pyruvate into mitochondria, glutamate fueling becomes vital in both cancers, as it helps to maximize lactate production through glycolysis and its further secretion. Elevated extracellular lactate concentration can be used to increase intracellular concentration in nearby cells in which lactate reduces to pyruvate to slow down terminal reactions of glycolysis in the case of nutrients deficiency, for example, in a case of tumor hypervascularization. However, lactate uptake will not lead to a decreasing glucose consumption rate nor replenish the pool of glycolysis intermediate metabolites, but it promotes processes that favor proliferation–PPP and amino acids synthesis from the intermediate metabolites. Thus, lactate excreted by one cell serves as fuel for other cancer cells to support biomass and energy production. In addition, a balanced level of intra- and extra-cellular lactate helps to protect cancer cells from apoptosis. The similarity of the genomic profile of cHCC-CCA to HCC and CCA profiles (Hong et al., 2026; Zhang Y. Z. et al., 2024) makes it natural to propose similar metabolic reprogramming in cHCC-CCA cells as in HCC and CCA, despite the lack of studies of specific glycolytic alterations in cHCC-CCA.

**TABLE 1:** Concise summary of metabolic alterations between HCC and CCA.

Segment	HCC features	CCA features
Glucose uptake	Increased GLUT family transporter expression, especially GLUT1 and GLUT3, correlates with tumor severity. Bidirectional transporter GLUT2 overexpression (if more pronounced than other family members) protects cells from substantially intensified glycolysis.	
Glycolysis	Increased Overexpression of HK2 and downstream enzymes, including PHGDH, ensures a high glycolytic rate and metabolite flux to support the PPP and the serine synthesis pathway.	
Lactate	Increased level Lactate is actively redistributed between cells to maintain a high level within tumor tissue, supporting mitochondrial activity and driving pyruvate flux into the TCA. High lactate levels also help to protect cancer cells from apoptosis and ferroptosis.	Increased production Significant overexpression of lactate transporters helps maintain a high glycolytic rate, while under lactic acidosis conditions, mitochondrial activity remains high. High extracellular lactate level protects cells from apoptosis and increases cells’ migration and invasion abilities.
Mitochondria activity	Reduced Vital for citrate efflux for further lipogenesis. Relies heavily on glutamine fueling.	Reduced Produce energy to increase the proliferation rate. Relies on glutamine for fueling, which helps maintain a high level of invasiveness.
Fatty acids pool replenishment	*De novo* synthesis Enhanced efflux of citrate and αKG from mitochondria ensures effective fatty acid synthesis, especially under hypoxic conditions.	Extracellular uptake *De novo* synthesis plays a negligible role and is activated only in later-stage tumors.
β-oxidation	Inhibited	Increased in highly-proliferative tumors only.
Lipid composition	Increased saturated and n9-monounsaturated FA levels in lipids of all classes, while the ratio and unsaturation degree of other unsaturated FA significantly decreased. SM levels increased, while ceramides are downregulated.	Increased TAG with reduced PC content reflects the necessity to accumulate FA pool in lipid droplets for further proliferation. Ceramides are upregulated, and SM are upregulated in highly proliferative tumors. No studies report specific data on fatty acid composition at this moment.

The metabolic alterations in cHCC-CCA tumors remain underexplored. The way lipid metabolism changed in cHCC-CCA cells can shed light on the specific processes underlying malignant transformation that lead to combined and mixed phenotypes in PLC cells.

In contrast to glycolysis, lipid metabolism differs between HCC and CCA (Table 1). *De novo* synthesis is a major source of fatty acids for lipogenesis in HCC. Reduced energy production through OXPHOS leads to a reduction in the number of mitochondria in HCC cells and a shift in mitochondrial activity from ATP production mainly to citrate production for further FA synthesis. Also, despite the upregulation of fatty acids-modifying enzymes, the overall unsaturation rate of FA decreases. The degree of essential fatty acids and their polyunsaturated derivatives decreases, while SFA and n-9MUFAs become upregulated. Thus, overall lipid composition alters both in phospholipids and TAGs due to the reduced availability of unsaturated FAs. In contrast to HCC, the uptake of extracellular FA plays an essential role in CCA, but a detailed investigation of FA and lipids composition of CCA has still not been carried out. Nevertheless, it is shown that FAO plays an essential role in energy production in highly proliferated CCA cells while being inhibited in HCC, where the expression of beta-oxidation-related enzymes negatively correlates with tumor size and aggressiveness. Thus, lipid metabolism appears to be a bifurcation point between two nosologies, and further investigation of lipid metabolism, especially in CCA and cHCC-CCA, is required to understand the pathogenesis of cHCC-CCA and improve diagnostics procedures and treatment strategies for PLC.

## Glossary

PLC: primary liver cancer
HCC: hepatocellular carcinoma
CCA: cholangiocarcinoma
cHCC-CCA: combined hepatocellular-cholangiocarcinoma
OXPHOS: oxidative phosphorylation
GLUT: glucose transporter
TCA: tricarboxylic acid cycle
PPP: pentose phosphate pathway
DRS: disease-related survival
iCCA: intrahepatic cholangiocarcinoma
DFS: disease-free survival
HIF-1α: hypoxia-inducible factor 1-alpha
EHD: extrahepatic bile duct cancer
18F-FDG: [18F]-2-fluoro-2-deoxy-d-glucose
OS: overall survival
RFS: recurrence-free survival
TNM: Tumor-Node-Metastasis
HK: hexokinase
G6P: glucose-6-phosphate
hCCA: hilar cholangiocarcinoma
NDC: non-dysplastic cirrhosis
LCD: liver cell dysplasia
F6P: fructose-6-phosphate
G6PD: glucose-6-phosphate dehydrogenase
PFK1: Phosphofructokinase 1
F1,6BP: fructose-1,6-bisphosphate
F2,6BP: fructose-2,6-bisphosphate
PFK2/F2,6BPase: 6-phosphofructo-2-kinase/fructose-2,6-bisphosphatase
ALDOA: aldolase A
PK: pyruvate kinase
G3P: D-glyceraldehyde 3-phosphate
DHAP: dihydroxyacetone phosphate
3PG: 3-phosphoglycerate
GSEA: Gene Set Enrichment Analysis
ALDOB: aldolase B
ETR: early tumor recurrence
PP2A: protein phosphatase 2A
p-Akt: phosphorylated Akt
GAPDH: glyceraldehyde-3-phosphate dehydrogenase
PGK1: phosphoglycerate kinase 1
LDHA: lactate dehydrogenase A
PHGDH: phosphoglycerate dehydrogenase
CARM1: coactivator-associated arginine methyltransferase 1
G2P: glycerol-2-phosphate
PARP14: anti-apoptotic protein poly(ADP-ribose) polymerase 14
HSP90: heat shock protein 90
GSK-3b: glycogen synthase kinase-3b
GTPBP4: guanosine triphosphate binding protein 4
EMT: epithelial-mesenchymal transition
FoxM1: Forkhead box M1
ROS: reactive oxygen species
LLA: lactic acidosis
THBS1: thrombospondin 1
LDHB: lactate dehydrogenas B
MCTs: monocarboxylate transporters
AFP: alpha fetoprotein
HCAR1: hydroxycarboxylic acid receptor 1
AMPK: AMP-activated protein kinase
SREBP1: sterol regulatory element-binding protein 1
SCD1: stearoyl-coenzyme A (CoA) desaturase-1
MUFAs: monounsaturated fatty acids
ACSL4: acyl-coenzyme A (CoA) synthetase long chain family member 4
PUFAs: polyunsaturated fatty acids
2PG: 2-phosphoglycerate
MPC: mitochondrial pyruvate carrier
PGC1α: peroxisome proliferator-activated receptor γ coactivator-1α
acetyl-CoA: acetyl-coenzyme A
PDC: pyruvate dehydrogenase complex
PDHE1a/PDHA1: E1a subunit of PDC
PDK: pyruvate dehydrogenase kinase
SSP: serine synthesis pathway
SIRT3: sirtuin-3, FASN, fatty acid synthase
αKG: α-ketoglutarate
2HG: 2-hydroxyglutarate
MMP9: matrix metalloproteinase 9
SDHB: succinate dehydrogenase B
GS: glutamine synthase
GLS: glutaminase
GLUD: glutamate dehydrogenase
TCGA: The Cancer Genome Atlas
CPTAC: Clinical Proteomic Tumor Analysis Consortium
FAO: fatty acid oxidation
FA: fatty acid
LCFAs: long-chain fatty acids
MCFAs: medium-chain fatty acids
SCFAs: short-chain fatty acids
CPT2: carnitine palmitoyltransferase-2
ACADM: medium-chain acyl-CoA dehydrogenase
CTP: citrate transport protein
PMCT: plasma membrane citrate transporter
ACC: acetyl-CoA carboxylase
ACLY: ATP citrate lyase
FATP5: fatty acid transport protein-5
FABPs: fatty acid-binding proteins
NHC: healthy cholangiocytes
BRD9: bromodomain-containing protein 9
LPL: lipoprotein lipase
SFA: saturated fatty acids
TAG: triacylglycerols
PC: phosphatidylcholine
PE: phosphoethanolamine
PI: phosphatidylinositol
SM: sphingomyelin
Cer: ceramide
PG: phosphatidylglycerol
PS: phosphatidylserine
CE: cholesterol ester
VLDL: very low-density lipoprotein
HDL: high-density lipoprotein.
